# Mortality Among Adults With Cancer Undergoing Chemotherapy or Immunotherapy and Infected With COVID-19

**DOI:** 10.1001/jamanetworkopen.2022.0130

**Published:** 2022-02-21

**Authors:** Csilla Várnai, Claire Palles, Roland Arnold, Helen M. Curley, Karin Purshouse, Vinton W. T. Cheng, Stephen Booth, Naomi A. Campton, Graham P. Collins, Daniel J. Hughes, Austin G. Kulasekararaj, Alvin J. X. Lee, Anna C. Olsson-Brown, Archana Sharma-Oates, Mieke Van Hemelrijck, Lennard Y. W. Lee, Rachel Kerr, Gary Middleton, Jean-Baptiste Cazier

**Affiliations:** 1 Centre for Computational Biology, University of Birmingham, Birmingham, United Kingdom; 2 Institute of Cancer and Genomic Sciences, University of Birmingham, Birmingham, United Kingdom; 3 Cancer Research UK Birmingham Centre, University of Birmingham, United Kingdom; 4 Edinburgh Cancer Research Centre, University of Edinburgh, Edinburgh, United Kingdom; 5 Leeds Institute of Medical Research, University of Leeds, Leeds, United Kingdom; 6 Oxford NIHR Biomedical Research Centre, Department of Haematology, Churchill Hospital, Oxford, United Kingdom; 7 Institute of Translational Medicine, Birmingham Health Partners, Birmingham, United Kingdom; 8 Department of Cancer Imaging, King’s College London, London, United Kingdom; 9 Department of Haematology, King’s College Hospital, London, United Kingdom; 10 UCL Cancer Institute, University College London, London, United Kingdom; 11 The Clatterbridge Cancer Centre, Wirral, United Kingdom; 12 The University of Liverpool, Liverpool, United Kingdom; 13 Institute of Inflammation and Ageing, University of Birmingham, Birmingham, United Kingdom; 14 Translational Oncology and Urology Research, School of Cancer and Pharmaceutical Sciences, King’s College London, London, United Kingdom; 15 Department of Oncology, Oxford University, Oxford, United Kingdom; 16 Institute of Immunology and Immunotherapy, University of Birmingham, Edgbaston, Birmingham, United Kingdom; 17 Queen Elizabeth Hospital, Birmingham, United Kingdom

## Abstract

**Importance:**

Large cohorts of patients with active cancers and COVID-19 infection are needed to provide evidence of the association of recent cancer treatment and cancer type with COVID-19 mortality.

**Objective:**

To evaluate whether systemic anticancer treatments (SACTs), tumor subtypes, patient demographic characteristics (age and sex), and comorbidities are associated with COVID-19 mortality.

**Design, Setting, and Participants:**

The UK Coronavirus Cancer Monitoring Project (UKCCMP) is a prospective cohort study conducted at 69 UK cancer hospitals among adult patients (≥18 years) with an active cancer and a clinical diagnosis of COVID-19. Patients registered from March 18 to August 1, 2020, were included in this analysis.

**Exposures:**

SACT, tumor subtype, patient demographic characteristics (eg, age, sex, body mass index, race and ethnicity, smoking history), and comorbidities were investigated.

**Main Outcomes and Measures:**

The primary end point was all-cause mortality within the primary hospitalization.

**Results:**

Overall, 2515 of 2786 patients registered during the study period were included; 1464 (58%) were men; and the median (IQR) age was 72 (62-80) years. The mortality rate was 38% (966 patients). The data suggest an association between higher mortality in patients with hematological malignant neoplasms irrespective of recent SACT, particularly in those with acute leukemias or myelodysplastic syndrome (OR, 2.16; 95% CI, 1.30-3.60) and myeloma or plasmacytoma (OR, 1.53; 95% CI, 1.04-2.26). Lung cancer was also significantly associated with higher COVID-19–related mortality (OR, 1.58; 95% CI, 1.11-2.25). No association between higher mortality and receiving chemotherapy in the 4 weeks before COVID-19 diagnosis was observed after correcting for the crucial confounders of age, sex, and comorbidities. An association between lower mortality and receiving immunotherapy in the 4 weeks before COVID-19 diagnosis was observed (immunotherapy vs no cancer therapy: OR, 0.52; 95% CI, 0.31-0.86).

**Conclusions and Relevance:**

The findings of this study of patients with active cancer suggest that recent SACT is not associated with inferior outcomes from COVID-19 infection. This has relevance for the care of patients with cancer requiring treatment, particularly in countries experiencing an increase in COVID-19 case numbers. Important differences in outcomes among patients with hematological and lung cancers were observed.

## Key Points

**Question:**

Is there an association between COVID-19 infection and mortality among adults with varying cancer types undergoing active treatment?

**Findings:**

In this cohort study of 2515 adult patients with cancer and COVID-19, hematological malignant neoplasms and lung cancer were associated with increased mortality. No association was found between recent treatment with chemotherapy and overall or COVID-19–specific mortality, and treatment with immunotherapy before COVID-19 diagnosis was associated with a significant reduction in mortality.

**Meaning:**

In this study, active systemic anticancer treatment was not associated with mortality in patients who also had COVID-19.

## Teaser

This cohort study evaluates whether systemic anticancer treatments, tumor subtypes, patient demographic characteristics, and comorbidities are associated with COVID-19 mortality.

## Introduction

More than 185 million people worldwide have had confirmed SARS-CoV-2. There have been 4 million COVID-19 infection–related deaths with more than 129 000 deaths in the UK alone.^1^ Early data suggested patients with cancer had a 2-fold higher risk of COVID-19–related mortality.^2,3^ As a result, cancer services made sweeping changes to treatment and follow-up strategies to minimize the risk of COVID-19 infection.

The UK Coronavirus Cancer Monitoring Project (UKCCMP) was launched on March 18, 2020. Early data identified that COVID-19 mortality in patients with cancer was primarily associated with age, sex, and comorbidities rather than recent systemic anticancer therapies (SACTs),^4^ a finding shared by other large prospective studies,^5^ and that tumor subtypes had differing mortality risks.^6^ We performed exploratory analyses aiming to determine whether mortality was associated with SACTs, tumor subtype, patient demographic characteristics (age and sex), and comorbidities using univariable and multivariable models in a large data set of patients with active cancer and with COVID-19 infection.

## Methods

### Data Collection and Study Design

Patient data were collected as previously described.^4,6^ Patients 18 years and older with active cancer and a clinical diagnosis of COVID-19 (98% had positive reverse transcription–polymerase chain reaction [RT-PCR] results) were eligible. Patients with active cancer were defined as those with metastatic disease, those undergoing active treatment, or those treated in the last 12 months. Patients were registered to this study by cancer centers when patients presented at hospital for either cancer treatment, which led to COVID-19 testing, or COVID-19 infection requiring treatment. Most data were collected and managed using REDCap electronic data capture tools^7,8^ hosted at the Institute of Translational Medicine, University of Birmingham, as described previously. For the 209 patients who tested positive for SARS-CoV-2 on RT-PCR at Guy’s and St Thomas’ Hospital and the 64 patients who tested positive at King’s College Hospital NHS Foundation Trusts, data were collected by their respective organizations.^9^ All 69 participating centers entered anonymized patient information. The information collected included ethnicity data, which was only entered if present in the patient’s medical record. Ethnicity information was collected following guidance from the UK government.^10^ Owing to the small number of patients with a recorded ethnicity other than White, the only analysis presented compared patients classified as any White background with those classified as any Black, Asian, or other minority ethnicity. Ethnicity data were collected because of early reports of worse outcomes in those infected with COVID-19 from Black, Asian, or minority ethnic backgrounds. All data were securely transferred into the Compute and Storage for Life Science infrastructure at the Centre for Computational Biology, University of Birmingham,^6^ and curated following a uniform annotation process.

Patients undergoing active treatment fewer than 4 weeks prior to their positive SARS-CoV-2 test were classified as under active treatment. The study period was March 18 to August 1, 2020, and as such all patients were unvaccinated. For further information see the eMethods in Supplement 1.

The UKCCMP was classified by the National Health Service Health Research Authority (HRA) as a public health surveillance project that did not require further ethical review or approval by the HRA.^11^ Each participating center provided confirmation that required local approvals were in place (including Caldicott approval if deemed necessary by each center). With HRA approval there was no requirement for individual patient consent. This report follows the Strengthening the Reporting of Observational Studies in Epidemiology (STROBE) reporting guideline for cohort studies.^12^

### Statistical Analysis

Overall, 2515 patients with valid outcome, age, sex, and comorbidity data were included in all analyses unless otherwise stated. eFigure 1 in Supplement 1 details why patients were excluded. The primary outcome of all-cause mortality was assessed by death during the primary hospitalization. A secondary outcome was COVID-19–specific death, for which COVID-19 was listed as a significant contributing factor within the same time frame.

Data processing, statistical analysis, and visualization were performed in R version 3.6.0 (R Project for Statistical Computing). Two-sided Welch *t* tests were used to compare continuous data; 2-sided Fisher exact tests were used to compare categorical data. Results are reported as statistically significant at the nominal threshold of *P* < .05. Power calculations were performed in R using the statmod package. Potential explanatory variables were analyzed using multivariable logistic regression adjusting for age, sex, and the presence of at least 1 of the following comorbidities: chronic kidney disease (CKD), chronic obstructive pulmonary disease (COPD), cardiovascular disease (CVD), diabetes, hypertension, or vascular diseases, as a binary variable. *P* values and 95% CIs were calculated with a sandwich estimator and cluster correction using the vcovCL() function of the sandwich R package (version 3.0-1) with the 69 participating centers as clusters and the HC1 bias correction. Nominal *P* values and 95% CIs are reported without mathematical correction for multiple comparisons.

## Results

Of the 2515 patients included in the study, 1464 (58%) were male, and the median (IQR) age was 72 (62-80) years. Overall, 1463 (58%) presented with at least 1 of the key comorbidities, and 272 (11%) were identified as Black, Asian, or another minority ethnic group.

A mortality rate of 38% was observed (966 patients); median (IQR) follow-up for all-cause death was 7 (3-13) days. A total of 1108 patients (44%) presented with mild symptoms of COVID-19, 701 (28%) with severe, 539 (21%) with critical, while 119 (5%) presented with no symptoms of COVID-19 (eTable 2 in Supplement 1). Overall, 493 of those who died (51%) presented with critical symptoms, compared with only 46 of those who survived (3%). A total of 131 patients (5%) were admitted to intensive care. The breakdown of characteristics for all patients, those receiving chemotherapy, those not receiving chemotherapy, and those not receiving any treatment can be found in eTable 2 in Supplement 1. Further information regarding cancer type, cancer stage, and COVID-19 severity are also provided.

We performed exploratory analyses to test the evidence to support associations of patient characteristics, tumor characteristics, and treatment types with outcomes in a large cohort of UK patients with cancer and a confirmed diagnosis of COVID-19. Male sex, older age, and presence of key comorbidities were associated with higher mortality (male sex: odds ratio [OR], 1.53; 95% CI, 1.23-1.90; older age: OR, 1.04; 95% CI, 1.03-1.05; presence of comorbidities: OR, 1.92; 95% CI, 1.57-2.34). The association between individual comorbidities and outcomes was also tested. When all patients were included, CKD and CVD were associated with higher all-cause mortality (CKD: OR, 1.58; 95% CI, 1.19-2.09; CVD: OR, 1.26; 95% CI, 1.03-1.55) (eTable 3 in Supplement 1). In patients with solid cancer, CKD, CVD, COPD, and diabetes were significantly associated with higher mortality, while in patients with hematological cancer, only CKD was significantly associated with higher mortality in multivariate analysis adjusting for age and sex only (OR, 1.81; 95% CI, 1.10-2.97). Higher BMI was also significantly associated with higher mortality (OR, 1.03; 95% CI, 1.004-1.07), as was smoking history (OR, 1.17; 95% CI, 1.03-1.34).

**Figure 1.**
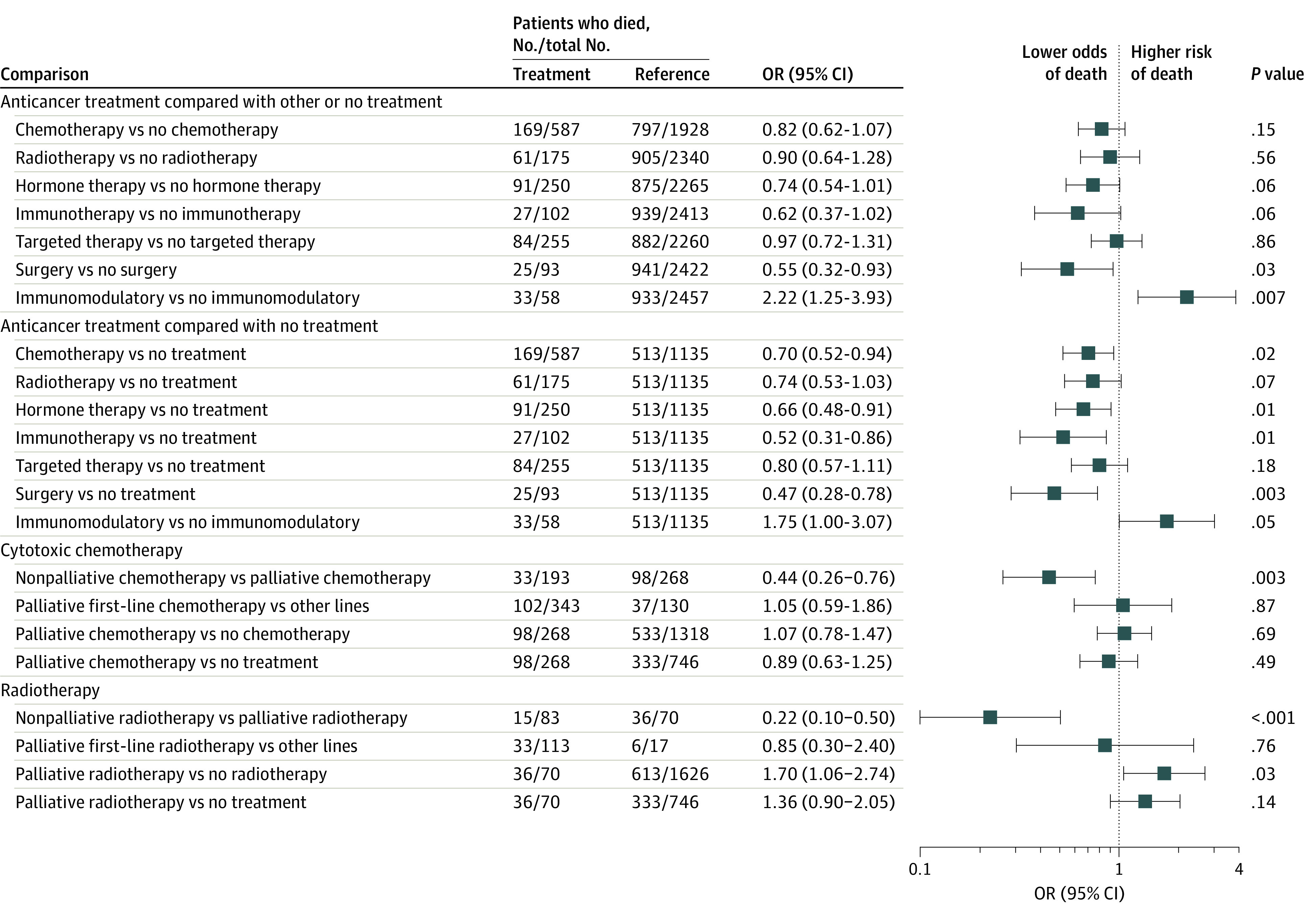
Association Between Anticancer Treatment Within 4 Weeks of COVID-19 Diagnosis and All-Cause Mortality Multivariate analyses, adjusted for age, sex, and comorbidities, are presented, and nominal *P* values are reported. OR indicates odds ratio.

We found no evidence that recent chemotherapy (among 587 participants) was associated with increased all-cause mortality compared with patients who had received other anticancer treatment (OR, 0.82; 95% CI, 0.62-1.07) or no anticancer treatment (OR, 0.70; 95% CI, 0.52-0.94) (Figure 1). We performed 2 sensitivity analyses, removing patients who were categorized as receiving surveillance or in remission (70 patients) and additionally removing those without cancer stage information (687 patients) to compare outcomes only in those with definitely active cancer. The results were consistent with those obtained with the total population (first analysis: OR, 0.67; 95% CI, 0.51-0.89; second analysis: OR, 0.52; 95% CI, 0.39-0.70). We confirmed the robustness of our results by comparing patients with recent chemotherapy with propensity score–matched patients (OR, 0.79; 95% CI, 0.62-1.02).

In an analysis of patients with solid cancer, chemotherapy (406 patients) was associated with lower all-cause mortality (chemotherapy vs no chemotherapy: OR, 0.61; 95% CI, 0.46-0.80; chemotherapy vs no anticancer treatment: OR, 0.48; 95% CI, 0.36-0.65). We previously reported an association between higher mortality and chemotherapy in patients with hematological cancer.^6^ In this larger data set of 604 patients and following reclassification of immunomodulatory treatments into a separate category (58 patients), 181 patients with hematological cancer had received recent chemotherapy. In this group, we no longer observed a statistically significant association between chemotherapy and mortality when adjusting for age, sex, and comorbidities (OR, 1.28; 95% CI, 0.85-1.95). Immunomodulatory treatment was associated with higher mortality (OR, 1.73; 95% CI, 1.00-2.99). The association remained in an analysis of only patients with myeloma (OR, 1.98; 95% CI, 1.06-3.69).

We tested the association between recent chemotherapy and patient outcome, stratified by cancer subtypes in multivariate analyses adjusted for age, sex (for non–sex-specific cancer types) and comorbidities. In the analysis of solid cancers, recent chemotherapy was associated with improved survival in patients with noncolorectal digestive organ (n = 67; OR, 0.32; 95% CI, 0.15-0.70), female genital organ (n = 41; OR, 0.25; 95% CI, 0.09-0.70), and breast (n = 84; OR, 0.36; 95% CI, 0.16-0.81) cancers. There was no association between recent chemotherapy and death for any individual hematological cancer type (eFigure 2 in Supplement 1). We also tested the association between recent chemotherapy and patient outcome, stratified by cancer stage in a multivariate analysis of patients with solid cancer. Receiving chemotherapy was associated with lower mortality for patients with primary cancers (759 patients; OR, 0.48; 95% CI, 0.30-0.77; *P* = .002) as well as for patients with metastatic cancers (868 patients; OR, 0.65; 95% CI, 0.45-0.93; *P* = .02).

Nonpalliative chemotherapy (neoadjuvant, adjuvant, or radical) was associated with lower mortality compared with palliative chemotherapy (33 of 193 [17.1%] vs 98 of 268 [36.6%]; OR, 0.44; 95% CI, 0.26-0.76) (Figure 1). However, there was no significant association between chemotherapy and all-cause mortality in an analysis of only patients receiving palliative care (Figure 1 and eTable 4 in Supplement 1). There was also no association between higher mortality and multimodal SACT or combination chemotherapy compared with single-agent chemotherapy (eTable 5 in Supplement 1).

We compared all-cause mortality of patients receiving other anticancer therapies in the 4 weeks before testing positive for COVID-19 with patients who were not receiving that anticancer therapy in the same time period (Figure 1 and eTable 4 in Supplement 1). Surgery (93 patients; OR, 0.55; 95% CI, 0.32-0.93) was associated with lower mortality compared with no surgery, while immunotherapy (102 patients; OR, 0.62; 95% CI, 0.37-1.02), hormonal therapy (250 patients; OR, 0.74; 95% CI, 0.54-1.01), radiotherapy (175 patients; OR, 0.90; 95% CI, 0.64-1.28), and targeted therapies (255 patients; OR, 0.97; 95% CI, 0.72-1.31) were not associated with higher mortality compared with no treatment with the same type of anticancer therapy. When compared with no cancer therapy, hormonal therapy (250 patients; OR, 0.66; 95% CI, 0.48-0.91) and immunotherapy (102 patients; OR, 0.52; 95% CI, 0.31-0.86) were significantly associated with lower mortality. We also observed an association between less severe COVID-19 symptoms and immunotherapy compared with no cancer treatment (OR, 0.63; 95% CI, 0.42-0.94). We explored the association between patient outcome and recent immunotherapy (<4 weeks) in patients with different cancer subtypes (eTable 6 in Supplement 1). While based on small numbers, no significant associations were observed in any single cancer type. In patients with lung cancer receiving check-point inhibitor (CPI) immunotherapy, chemotherapy, combination chemotherapy and immunotherapy, targeted treatment, or radiotherapy in the 4 weeks before a COVID-19 diagnosis were not associated with mortality compared with no treatment with these therapies (eTable 7 in Supplement 1).

A total of 329 patients received their last cancer treatment less than 3 weeks before their COVID-19 diagnosis; 116 patients, within 3 to 6 weeks; and 86 patients, within 6 to 12 weeks. In multivariate analysis, the timing of recent cancer treatment was not associated with mortality. Cancer treatment less than 3 weeks before COVID-19 diagnosis was not associated with mortality compared with patients treated 3 to 12 weeks before diagnosis (OR, 0.85; 95% CI, 0.58-1.23) (eTable 8 in Supplement 1).

**Figure 2.**
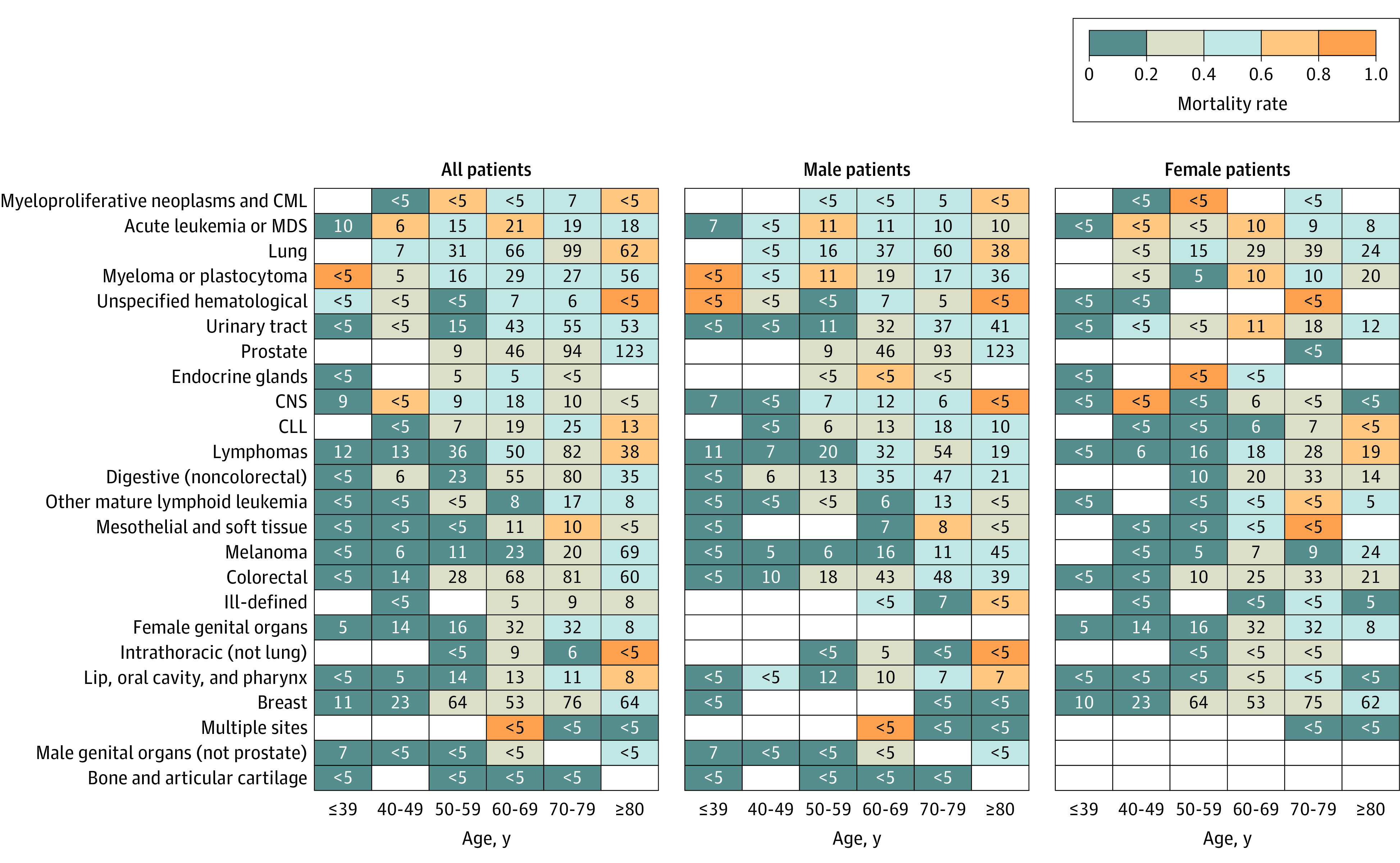
All-Cause Case Mortality Rate After Presentation With COVID-19, by Cancer Type, Age, and Sex Sorted by Decreasing Overall Rate Cells are colored by case mortality rate; numbers in cells are the number of patients with the displayed sex, age, and cancer type combination, with values for cells including fewer than 5 participants suppressed to protect patient privacy. Empty cells indicate no patients. CLL indicates chronic lymphocytic leukemia; CML, chronic myeloid leukemia; CNS, central nervous system; and MDS, myelodysplastic syndrome.

Acute leukemia and myelodysplastic syndrome (MDS) were significantly associated with higher mortality (OR, 2.16; 95% CI, 1.30-3.60) (Table). Patients with myeloma or plasmacytoma under the age of 60 years had particularly high mortality rates (12 of 22 [54.5%]) (Figure 2), and these cancers were associated with higher mortality (OR, 1.53; 95% CI, 1.04-2.26). Lung cancer was also significantly associated with higher mortality (265 patients; OR, 1.58; 95% CI, 1.11-2.25) (Table), with similar effect sizes observed in patients with small cell lung cancer and patients with non–small cell lung cancers. Neither COPD (86 of 265 patients; OR, 1.25; 95% CI, 0.71-2.21) nor smoking history (175 of 201 patients; OR, 0.46; 95% CI, 0.16-1.30) were associated with a higher mortality in patients with lung cancer. Consistent with other studies, no significant association between cytotoxic chemotherapy or immunotherapy was observed in patients with lung cancer.^13,14,15^

**Table.** Association Between Cancer Type and All-Cause Mortality Following COVID-19 Diagnosis Compared With Patients with Noncolorectal, Digestive Organ Cancer

Cancer subtype (*ICD-10* code)	Mortality rate	Patients, No.		Univariate analysis		Multivariate analysis, adjusted for age and sex		Multivariate analysis, adjusted for age, sex, and comorbidities	
		With the cancer type	With the cancer type who died	OR (95% CI)	*P* value	OR (95% CI)	*P* value	OR (95% CI)	*P* value
Myeloproliferative neoplasms and CML (C92.1, D45-D47)	0.529	17	9	1.87 (0.61-5.83)	.30	2.04 (0.79-5.29)	.14	2.00 (0.75-5.32)	.16
Acute leukemia or MDS (C91.0, C92.0, D46)	0.506	89	45	1.70 (1.00-2.91)	.04	2.11 (1.25-3.54)	<.001	2.16 (1.30-3.60)	<.001
Lung (C34)	0.494	265	131	1.63 (1.10-2.41)	.01	1.63 (1.13-2.34)	<.001	1.58 (1.11-2.25)	.01
Myeloma/plasmacytoma (C.90)	0.493	134	66	1.62 (1.01-2.58)	.04	1.53 (1.04-2.24)	.03	1.53 (1.04-2.26)	.03
Hematological cancer, other/unspecified	0.478	23	11	1.52 (0.58-3.99)	.37	1.70 (0.66-4.42)	.27	1.88 (0.72-4.94)	.20
Urinary tract (C64-C68)	0.453	172	78	1.38 (0.89-2.14)	.14	1.25 (0.77-2.01)	.37	1.23 (0.76-1.97)	.39
Prostate (C61)	0.43	272	117	1.26 (0.85-1.86)	.26	0.72 (0.43-1.20)	.20	0.69 (0.42-1.15)	.16
Endocrine glands (C73-C75)	0.429	14	6	1.25 (0.34-4.29)	.78	1.55 (0.39-6.18)	.54	1.62 (0.39-6.71)	.50
CNS (C69-C72)	0.404	52	21	1.13 (0.57-2.20)	.75	1.63 (0.81-3.26)	.17	1.66 (0.84-3.31)	.15
CLL (C91.1)	0.397	68	27	1.10 (0.60-2.00)	.77	1.04 (0.53-2.02)	.91	1.07 (0.54-2.11)	.84
Lymphomas (C81-C85, C88)	0.385	231	89	1.04 (0.69-1.57)	.84	1.15 (0.73-1.81)	.55	1.16 (0.74-1.84)	.51
Other mature lymphoid leukemias (C96)	0.357	42	15	0.93 (0.43-1.94)	.86	0.93 (0.48-1.79)	.82	0.89 (0.45-1.74)	.73
Mesothelial and soft tissue (C45-C49)	0.355	31	11	0.92 (0.37-2.14)	>.99	0.96 (0.40-2.33)	.93	0.96 (0.39-2.35)	.93
Melanoma (C43-C44)	0.354	130	46	0.91 (0.56-1.48)	.73	0.60 (0.36-1.00)	.05	0.59 (0.35-1.00)	.05
Colorectal (C18-C21)	0.34	253	86	0.86 (0.57-1.29)	.49	0.84 (0.55-1.28)	.42	0.84 (0.55-1.28)	.41
Ill-defined (C76-C80)	0.304	23	7	0.73 (0.24-1.98)	.65	0.62 (0.27-1.43)	.27	0.67 (0.29-1.55)	.35
Female genital organs (C51-C58)	0.299	107	32	0.71 (0.41-1.21)	.21	1.43 (0.79-2.58)	.24	1.45 (0.79-2.64)	.23
Respiratory and intrathoracic organs (not lung; C30-C33, C35-C39)	0.278	18	5	0.64 (0.17-2.02)	.46	0.73 (0.28-1.92)	.52	0.72 (0.28-1.86)	.50
Lip, oral cavity, and pharynx (C00-C14)	0.273	55	15	0.63 (0.30-1.25)	.20	0.76 (0.38-1.51)	.43	0.78 (0.39-1.54)	.47
Breast (C50)	0.244	291	71	0.54 (0.36-0.81)	<.001	0.80 (0.49-1.30)	.37	0.85 (0.52-1.40)	.53
Male genital organs, not prostate (C60, C62-C63)	0.133	15	2	0.26 (0.03-1.19)	.09	0.40 (0.08-1.93)	.26	0.40 (0.08-1.89)	.25
Bone and articular cartilage (C40-C41)	0	6	0	0 (0-1.46)	.09	0 (0 to ∞)	NA	0 (0 to ∞)	NA
Digestive organs, noncolorectal (C15-C17, C22-C26)	0.375	200	75	1 [Reference]	NA	1 [Reference]	NA	1 [Reference]	NA

Abbreviations: CLL, chronic lymphocytic leukemia; CML, chronic myeloid leukemia; CNS, central nervous system; *ICD-10*, *International Statistical Classification of Diseases and Related Health Problems, Tenth Revision*; MDS, myelodysplastic syndrome; NA, not applicable; OR, odds ratio.

Blood indices were investigated for an association with patient outcome (eTable 9 in Supplement 1). C-reactive protein levels greater than the median value, neutrophilia, lymphopenia, and neutrophil-to-lymphocyte ratio were significantly associated with higher mortality. Neutropenia (neutrophils <2000/μL [to convert to cells × 10^9^ per liter, multiply by 0.001]) was not significantly associated with higher mortality.

## Discussion

This UKCCMP data set provides a unique opportunity to investigate a population within 1 health care system, the NHS. The data will facilitate better risk stratification of patients with cancer who may be exposed to COVID-19 and will permit clinicians to devise individualized care plans with patients that minimize disruption to cancer care.^16^ Overall, 38% of patients in UKCCMP died, and despite 49% of patients presenting with severe or critical COVID-19 illness, only 5% of patients were treated on an intensive care unit (eTable 10 in Supplement 1). CVD and CKD were associated with higher mortality across all patients. These data suggest patients with cancer, COVID-19, and known comorbidities represent a particularly vulnerable group. Cancer teams need to work closely with intensivists and primary and secondary care teams to ensure patients with cancer are offered the appropriate level of treatment.^17^

We and other large studies did not find an association between SACT and mortality.^4,5^ In this expanded cohort, we again found no association between anticancer treatment and all-cause mortality in patients with solid or hematological malignant neoplasms. In the combined analysis of solid and hematological cancers and the association between chemotherapy and mortality, the study had 80% power to detect an effect size (OR) of 1.2 or greater. An analysis based on propensity score matching confirmed the results identified using the regression models presented in the Results section. Consistent with the lack of excess mortality associated with recent chemotherapy, we did not observe a significant association between neutropenia and mortality.

Our findings regarding chemotherapy are at odds with a recent report from CCC19,^18^ which identified an association between recent chemotherapy and higher mortality, a finding with potentially significant policy and health care implications. Unlike the UKCCMP cohort that comprises only active cancers, only 61% of the CCC19 cohort had cancer that was present, active, or treated within the past year (eTable 11 in Supplement 1). The remaining 49% were in remission or had no evidence of disease. Furthermore, unlike the UKCCMP, missing clinical variables were imputed for more than 10% of hospitalized patients. Those without an active cancer diagnosis were included in the CCC19 analysis of the associations between chemotherapy and patient outcome. The outcomes of patients with historical cancers and COVID-19 infection are likely to be very different from those with active cancer. When we performed a sensitivity analysis removing 70 patients (2.8%) in UKCCMP categorized as being on surveillance or in remission, the association of recent chemotherapy and lower mortality became significant.

Immunotherapy (principally CPI), hormonal therapy, targeted therapy, radiotherapy, or surgery within 4 weeks of COVID-19 diagnosis were also not associated with higher mortality. To our knowledge, this is the first study to demonstrate significant associations between treatment with CPIs (used to treat melanoma as well as lung and renal cancer) and lower mortality and less severe COVID-19 symptoms. It may be that the association between recent CPI and improved outcome may be explained by enhanced antiviral T-cell immunity. Further information on the association of CPI with antiviral immunity and T-cell exhaustion is needed.

Conversely, we identified an association between higher mortality and immunomodulatory drugs lenalidomide, thalidomide, and pomalidomide used only to treat patients with myeloma, who are known to have increased mortality following COVID-19 infection. The association remained in an analysis of only patients with myeloma (OR, 1.98; 95% CI, 1.06-3.69). These findings need urgent validation in other data sets and are important given suggestions that their use might temper the inflammatory storm that drives severe COVID-19.^19^ It is necessary to understand the association of these drugs with antiviral immunity and efficacy of vaccination is also needed.

Our data confirms the association between higher mortality and hematological malignant neoplasms, consistent with UK primary care data.^20^ The COVID-19 immunological signature and postviral clearance immune state of patients with solid cancer is similar to the signature of people with COVID-19 infection but without cancer.^21^ In contrast, patients with hematological cancer and COVID-19 have much less immune activation, high levels of CD8^+^ T-cell exhaustion, severe B cell cytopenia. and inconsistent antibody responses. The only solid cancer in our data set associated with significantly worse outcome was lung cancer. COVID-19 infection primarily affects the lung, and lung cancer often occurs in the setting of chronic tobacco smoke–mediated damage and reduced respiratory reserve. We reported in the pre-COVID era a significantly higher mortality rate in patients with lung cancer hospitalized with pneumonia compared with patients with other cancers.^22^ COPD was associated with higher mortality in patients with solid cancer, a comorbidity very strongly associated with lung cancer. While COPD and smoking were not significantly associated with COVID-19–related mortality in patients with lung cancer, COPD is often underdiagnosed.^23,24^ In the Thoracic Cancers International COVID-19 Collaboration (TERAVOLT) study, most cases (80%) had a positive smoking history, which, along with age 65 years and older, high cancer stage, Eastern Cooperative Oncology Group performance status of 2 or greater and prior steroid use was associated with increased mortality.^22^ Overall, 87% of the patients with lung cancer in this study who had available smoking history had current or former smoking habits. Smoke exposure has been associated with increased angiotensin-converting enzyme 2 expression, a key component of SARS-CoV-2 cell binding and entry.^25,26^ Importantly, however, we found no significant association between death and cytotoxic chemotherapy or immunotherapy in patients with lung cancer, consistent with other studies.^13,14,15^

### Limitations

This study has limitations. Our results highlight observations that are potentially important for clinical practice in a well-powered study of 2515 patients with active cancer. We have highlighted the nominally significant results obtained given the exploratory nature of our analysis. However, it should be noted that none of the associations observed between treatment and mortality would remain significant after multiple testing correction, with the exception of the analysis of palliative vs nonpalliative radiotherapy. The median follow-up of patients in this cohort was short, and our results did not consider patients who may have been discharged and then subsequently died. We do, however, expect that if there were such events, there would only be a small number that the cancer centers reporting to us would not have been aware of. Performance status was not collected for this cohort. This is a serious limitation, but the impact is mitigated as much as possible by having information on patient comorbidities and adjusting for these in all analyses presented.

## Conclusions

In this study, while patients with cancer had poorer COVID-19 outcomes than other individuals with COVID-19,^2,3^ such difference in outcome may be associated with age, sex, comorbidities, and cancer subtype rather than anticancer treatments. Recent chemotherapy was not associated with all-cause mortality, and recent immunotherapy was associated with less severe COVID-19 symptoms and lower mortality.

## Notes

Supplement 1. **eMethods.** Data Processing **eFigure 1.** Selection Steps for our Cohort **eFigure 2.** Association of Chemotherapy With All-Cause Death For Individual Cancer Types **eTable 1.** Drug Classification of Systemic Anticancer Therapy **eTable 2. **Basic Patient Information Within 4 Categories: All, Patients Receiving Chemotherapy, Patients Not Receiving Chemotherapy (Including Not Receiving Treatment), and Patients Not Receiving Treatment **eTable 3. **Association of Comorbidities on Patient Outcome for All Patients, All-Cause Death to Survive (Including Remaining in Hospital), Using Patients With Valid Outcome, Age, Sex, and Comorbidities **eTable 4.** Association of Anticancer Treatment in the 4 Weeks Prior to COVID-19 Diagnosis With All-Cause Mortality **eTable 5. **Association of Multiple Treatment Types on All-Cause Mortality **eTable 6.** Association of Recent Immunotherapy (in the 4 Weeks Before a COVID-19 Diagnosis) With All-Cause Mortality for Individual Cancer Types **eTable 7.** Association of Cancer Treatments With All-Cause Mortality for Patients With Lung Cancer **eTable 8.** Association of the Time Difference Between the Last Cancer Treatment and COVID-19 Diagnosis With All-Cause Mortality **eTable 9.** Association of Blood Count Levels of CRP, Neutrophils, and Lymphocytes With All-Cause Mortality **eTable 10.** Patient Information on Admission to Intensive Care Unit **eTable 11.** Comparison of the Patients Included in the CCC19 Cohort [3] and This Study (UKCCMP)
eReferences.

Supplement 2.
UKCCMP Team Members
