# Error in Supplement 2

**DOI:** 10.1001/jamanetworkopen.2022.10276

**Published:** 2022-04-08

In the Original Investigation titled “Mortality Among Adults With Cancer Undergoing Chemotherapy or Immunotherapy and Infected With COVID-19,”^1^ published February 21, 2022, there was an error in a nonauthor collaborator’s name in Supplement 2. This article has been corrected.^1^
