# Supplementary material for: Mortality Among Adults With Cancer Undergoing Chemotherapy or Immunotherapy and Infected With COVID-19
Source: JAMA Netw Open. 2022 Feb 21;5(2):e220130. doi: 10.1001/jamanetworkopen.2022.0130 (PMC8861846; doi:10.1001/jamanetworkopen.2022.0130)
Supplement: Supplement 1. — eMethods. Data Processing eFigure 1. Selection Steps for our Cohort eFigure 2. Association of Chemotherapy With All-Cause Death For Individual Cancer Types eTable 1. Drug Classification of Systemic Anticancer Therapy eTable 2. Basic Patient Information Within 4 Categories: All, Patients Receiving Chemotherapy, Patients Not Receiving Chemotherapy (Including Not Receiving Treatment), and Patients Not Receiving Treatment eTable 3. Association of Comorbidities on Patient Outcome for All Patients, All-Cause Death to Survive (Including Remaining in Hospital), Using Patients With Valid Outcome, Age, Sex, and Comorbidities eTable 4. Association of Anticancer Treatment in the 4 Weeks Prior to COVID-19 Diagnosis With All-Cause Mortality eTable 5. Association of Multiple Treatment Types on All-Cause Mortality eTable 6. Association of Recent Immunotherapy (in the 4 Weeks Before a COVID-19 Diagnosis) With All-Cause Mortality for Individual Cancer Types eTable 7. Association of Cancer Treatments With All-Cause Mortality for Patients With Lung Cancer eTable 8. Association of the Time Difference Between the Last Cancer Treatment and COVID-19 Diagnosis With All-Cause Mortality eTable 9. Association of Blood Count Levels of CRP, Neutrophils, and Lymphocytes With All-Cause Mortality eTable 10. Patient Information on Admission to Intensive Care Unit eTable 11. Comparison of the Patients Included in the CCC19 Cohort [3] and This Study (UKCCMP) eReferences. [file jamanetwopen-e220130-s001.pdf]

Supplemental Online Content

Várnai C, Palles C, Arnold R, et al; UKCCMP Team. Mortality among adults with cancer undergoing chemotherapy or immunotherapy and infected with COVID-19. *JAMA Netw Open*. 2022;5(2):e220130. doi:10.1001/jamanetworkopen.2022.0130

eMethods. Data Processing
eFigure 1. Selection Steps for our Cohort
eFigure 2. Association of Chemotherapy With All-Cause Death For Individual Cancer Types
eTable 1. Drug Classification of Systemic Anticancer Therapy
eTable 2. Basic Patient Information Within 4 Categories: All, Patients Receiving Chemotherapy, Patients Not Receiving Chemotherapy (Including Not Receiving Treatment), and Patients Not Receiving Treatment
eTable 3. Association of Comorbidities on Patient Outcome for All Patients, All-Cause Death to Survive (Including Remaining in Hospital), Using Patients With Valid Outcome, Age, Sex, and Comorbidities
eTable 4. Association of Anticancer Treatment in the 4 Weeks Prior to COVID-19 Diagnosis With All-Cause Mortality
eTable 5. Association of Multiple Treatment Types on All-Cause Mortality
eTable 6. Association of Recent Immunotherapy (in the 4 Weeks Before a COVID-19 Diagnosis) With All-Cause Mortality for Individual Cancer Types
eTable 7. Association of Cancer Treatments With All-Cause Mortality for Patients With Lung Cancer
eTable 8. Association of the Time Difference Between the Last Cancer Treatment and COVID-19 Diagnosis With All-Cause Mortality
eTable 9. Association of Blood Count Levels of CRP, Neutrophils, and Lymphocytes With All-Cause Mortality
eTable 10. Patient Information on Admission to Intensive Care Unit
eTable 11. Comparison of the Patients Included in the CCC-19 Cohort [3] and This Study (UKCCMP)
eReferences.

This supplemental material has been provided by the authors to give readers additional information about their work.

© 2022 Várnai C et al. *JAMA Network Open.*

eMethods. Data Processing

Tumour types were classified according to International Classification of Diseases, 10th Revision (ICD-10) codes (WHO)[1]. Severity of COVID-19 was classified according to World Health Organization (WHO) guidelines[2]. Anticancer treatments received <4 weeks prior to testing positive for SARS-CoV-2 were categorized by the participating sites as chemotherapy, radiotherapy, immunotherapy, targeted therapy, or ‘other treatment’, with multiple options possible. Thalidomide, lenalidomide and pomalidomide were classified as immunomodulatory drugs. Free text information on drugs administered was curated by an expert team into treatment categories (eTable 1 in the Supplement). The resulting mapping information (drug to treatment type) was then programmatically applied to the data, resulting in a consistent mapping of individual drugs to treatment regimens. Main comorbidity types assigned by the participating sites using a controlled input vocabulary were unified with curated additional free text information into the following categories: chronic kidney disease (CKD), chronic obstructive pulmonary disease (COPD), cardiovascular disease (CVD), diabetes (DM), hypertension (HTN), vascular diseases, and ‘other’. Patients with no comorbidities reported, but not explicitly reported as having no comorbidities, were assigned to have no comorbidities (344 patients). All curation resulted in additional data columns and all initially entered data has not been changed to avoid accidental corruption of the data, and no cancer types have been removed from the core analyses.

Patient weight and height was standardized to kg and cm respectively and body mass index (BMI) calculated, or set to ‘not available’ for clear input errors and missing data.
The treatment type immunotherapy was assigned to patients receiving checkpoint inhibitor (CPI) immunotherapy only. Four patients on Bacillus Calmette-Guerin (BCG) treatment were categorized as on “other” treatment. The treatment types radiotherapy and surgery, which are independent of administered drugs, were retained as entered by the participating center. If no drug name was provided but treatment type information was available e.g. chemotherapy, the data has been used. If the free text information indicated that treatment occurred prior to 4 weeks before the positive SARS-CoV-2 RT-PCR test, the entry has been set to ‘not on treatment’. Patients on supportive drugs only were marked as ‘not on anticancer treatment’.
C-reactive protein (CRP) levels from patients’ blood counts were recorded initially (before the 21st May) as high or low (using 1 or 0), as opposed to the precise count later recorded. CRP data captured in this way, for 200 patients was excluded.

eFigure 1. Selection Steps for our Cohort

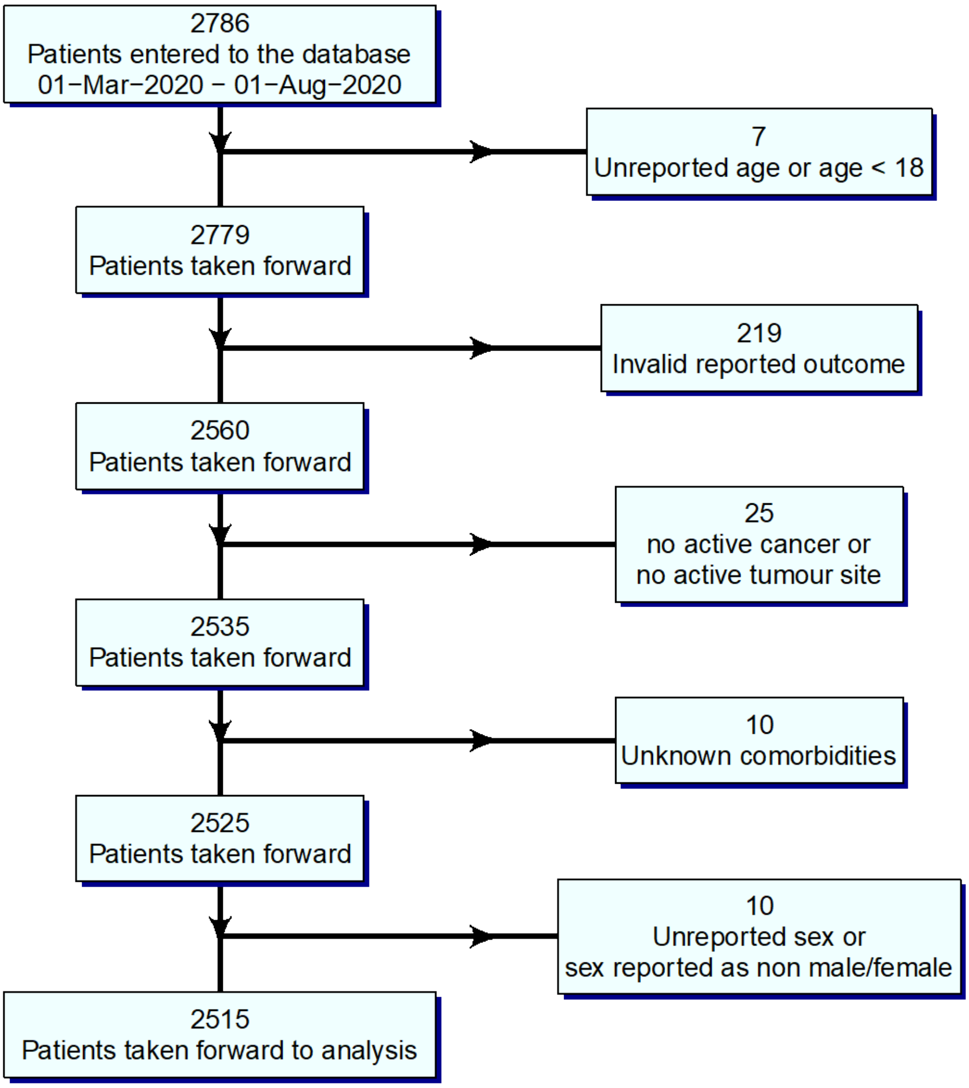

© 2022 Várnai C et al. *JAMA Network Open.*

eFigure 2. Association of Chemotherapy With All-Cause Death For Individual Cancer Types

Cancer subtype	Chemo Died/All	Reference Died/All	Odds ratio for death (95% CI)	P value	Fatality
Male genital organs (not prostate) (C60, C62−C63)	0/5	2/10	0 (0−0)	2.8e−16	0
Lip, oral cavity and pharynx	1/17	14/38	0.24 (0.02−3.16)	0.28	0.059
Breast (C50)	8/84	63/207	0.36 (0.16−0.81)	0.013	0.095
Female genital organs (C51−C58)	5/41	27/66	0.25 (0.09−0.7)	8.5e−03	0.122
Ill−defined (C76−C80)	1/6	6/17	0.33 (0.02−5.53)	0.443	0.167
Digestive organs (non colorectal) (C15−C17, C22−C26)	13/67	62/133	0.32 (0.15−0.7)	4.6e−03	0.194
Respiratory and intrathoracic organs (not lung; C30−C33, C35−C39)	1/5	4/13	0.68 (0.04−13.3)	0.802	0.2
Colorectal (C18−C21)	17/75	69/178	0.68 (0.34−1.36)	0.272	0.227
Mesothelial and soft tissue (C45−C49)	2/8	9/23	0.46 (0.06−3.66)	0.46	0.25
CNS (C69−C72)	4/14	17/38	0.61 (0.15−2.51)	0.492	0.286
Lymphomas_WM	34/92	55/139	1.07 (0.59−1.91)	0.831	0.37
OtherMatureLymphoidLeukaemia	3/8	12/34	0.69 (0.08−5.96)	0.739	0.375
Urinary tract (C64−C68)	5/12	73/160	0.87 (0.25−3.09)	0.832	0.417
Lung (C34)	23/52	108/213	0.82 (0.43−1.54)	0.531	0.442
ALL_AML_MDS	23/46	22/43	1.19 (0.39−3.58)	0.763	0.5
other haematological	2/4	9/19	1.34 (0.15−12.12)	0.796	0.5
Prostate (C61)	7/13	110/259	2.43 (0.88−6.75)	0.087	0.538
Myeloma_plasmacytoma	12/22	54/112	1.32 (0.45−3.84)	0.615	0.545
CLL	3/5	24/63	7.09 (1.19−42.22)	0.031	0.6
Endocrine glands (C73−C75)	2/3	4/11	1.46 (0.04−56.8)	0.838	0.667
MPN_CML	3/4	6/13	6.23 (0.02−1581)	0.517	0.75

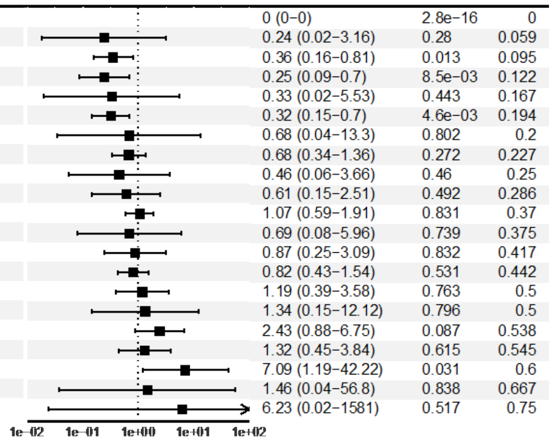

© 2022 Várnai C et al. *JAMA Network Open.*

eTable 1. Drug Classification of Systemic Anticancer Therapy

Drug names patients received in the 4 weeks before testing positive for COVID-19 and how they were categorized for analysis of the impact of SACT. Where possible drug regimens have been split apart into the drugs that make up the regimen. Patients only receiving supportive treatment were considered to be not on treatment in the 4 weeks prior to COVID-19 diagnosis.

Drug name	Drug classification used in UKCCMP
Abemaciclib	Targeted Therapy
Abiraterone	Hormone Therapy and steroid
Abraxane	Chemotherapy
Adcal D	Supportive Treatment
Adrenal Deprivation Therapy	Hormone Therapy
Adriamycin	Chemotherapy
Afatinib	Targeted Therapy
Alectinib	Targeted Therapy
Anastrozole	Hormone Therapy
Anti PD-1	Immunotherapy
Anti CTLA-4	Immunotherapy
Anti PD L-1	Immunotherapy
Atezolizumab	Immunotherapy
Dacarbazine	Chemotherapy
Bleomycin	Chemotherapy
Axitinib	Targeted Therapy
Azacitidine	Chemotherapy
Azathioprine	Other Therapy
Bacillus Calmette Gu Rin	Other Therapy
Baxarotene IFN Alpha Photopharesis	Other Therapy
Bentuzimab	Targeted Therapy
Bevacizumab	Targeted Therapy
Bicalutamide	Hormone Therapy
Binimetinib	Targeted Therapy
Bisphosphonate	Supportive Treatment
Bleomycin	Chemotherapy
Bortezomib	Targeted Therapy
Bosutinib	Targeted Therapy
Brentuximab	Targeted Therapy
Busulfan	Chemotherapy
Cabazitaxel	Chemotherapy
Cabozantinib	Targeted Therapy
Capecitabine	Chemotherapy
Carbazitaxel	Chemotherapy

© 2022 Várnai C et al. *JAMA Network Open.*

Carboplatin	Chemotherapy
Carfilzomib	Targeted Therapy
Carmustine	Chemotherapy
Ceritinib	Targeted Therapy
Cetuximab	Targeted Therapy
Chemoembolization	Other Therapy
Chlorambucil	Chemotherapy
Cisplatin	Chemotherapy
Cladribine	Chemotherapy
Crizotinib	Targeted Therapy
Cyclophosphamide	Chemotherapy
Cytarabine	Chemotherapy
Dabrafenib	Targeted Therapy
Dacarbazine	Chemotherapy
Daratumumab	Targeted Therapy
Darbepoetin Filgrastim	Supportive Treatment
Dasatinib	Targeted Therapy
Daunorubicin	Chemotherapy
Debrafenib	Targeted Therapy
Decapeptyl	Hormone Therapy
Degarelix	Hormone Therapy
Denosumab	Supportive Treatment
Dexamethasone	Steroid
Docetaxel	Chemotherapy
Doxorubicin	Chemotherapy
Durvulumab	Immunotherapy
Encorafenib	Targeted Therapy
Enzalutamide	Hormone Therapy
Epirubicin	Chemotherapy
EPO	Supportive Treatment
EPO GCSF	Supportive Treatment
Eribulin	Chemotherapy
Erlotinib	Targeted Therapy
Etoposide	Chemotherapy
Everolimus	Targeted Therapy
Exemestane	Hormone Therapy
Extracorporeal Photophoresis	Other Therapy
Fludarabine	Chemotherapy
Fluorouracil	Chemotherapy
Fulvestrant	Hormone Therapy
Gefitinib	Targeted Therapy

© 2022 Várnai C et al. *JAMA Network Open.*

Gemcitabine	Chemotherapy
Gen Bispecific Antibody	Targeted Therapy
Glivec	Targeted Therapy
Goserelin	Hormone Therapy
Guadecitabine	Chemotherapy
Hydroxycarbimide	Chemotherapy
Ibrutinib	Targeted Therapy
Idarubicin	Chemotherapy
Ifosfomide	Chemotherapy
Imatinib	Targeted Therapy
Inotuzumab	Targeted Therapy
Ipatasertib	Targeted Therapy
Ipilmumab	Immunotherapy
Irinotecan	Chemotherapy
Isatuximab	Targeted Therapy
Ixabepilone	Targeted Therapy
Ixazomib	Targeted Therapy
Jakal	Targeted Therapy
Lanreotide	Targeted Therapy
Lenolidamide	Immunomodulatory
Letrozole	Hormone Therapy
Leuprorelin	Hormone Therapy
Levantinib	Targeted Therapy
Levetiracetam	Other Therapy
Luteinizing Hormone-Releasing Hormone Agonist	Hormone Therapy
Lolatinib	Targeted Therapy
Lomustine	Chemotherapy
Lonsurf	Chemotherapy
Lorlatinib	Targeted Therapy
Mecaptopurine	Chemotherapy
Melphalan	Chemotherapy
MEPFAC Trial	Supportive Treatment
Mercaptopurine	Chemotherapy
Methotrexate	Chemotherapy
Midostaurin	Targeted Therapy
Mitomycin	Chemotherapy
Mogamulizumab	Targeted Therapy
Monoclonal	Targeted Therapy
Mylotarg	Chemotherapy
Nelarabine	Chemotherapy

© 2022 Várnai C et al. *JAMA Network Open.*

Neratinib	Targeted Therapy
Nintedanib	Targeted Therapy
Nivolumab	Immunotherapy
Octreotide	Hormone Therapy
Olaparib	Targeted Therapy
Osimertinib	Targeted Therapy
Oxaliplatin	Chemotherapy
Paclitaxcel	Chemotherapy
Paclitaxcel Albumin	Chemotherapy
Palbociclib	Targeted Therapy
Panitumumab	Targeted Therapy
Panobinostat	Targeted Therapy
Pazopanib	Targeted Therapy
Pembrolizumab	Immunotherapy
Pemetrexed	Chemotherapy
Pentostatin Monotherapy	Chemotherapy
Pertuzumab	Targeted Therapy
Polatuzumab	Targeted Therapy
Pomalidomide	Immunomodulatory
Prednisolone	Steroid
Procarbazine	Chemotherapy
PROSTAP	Hormone Therapy
Proteasome Inhibitor	Targeted Therapy
PROVERA	Hormone Therapy
Raltitrexed	Chemotherapy
Ribociclib	Targeted Therapy
Rucaparib	Targeted Therapy
Ruxolitinib	Targeted Therapy
Sorafenib	Targeted Therapy
Streptozocin	Chemotherapy
Sunitinib	Targeted Therapy
SYD985	Targeted Therapy
TACE	Other Therapy
Tamoxifen	Hormone Therapy
Tamsulosin	Other Therapy
Taxotere	Chemotherapy
Temozolomide	Chemotherapy
Thalidomide	Immunomodulatory
Thiotepa	Chemotherapy
Tivozantinib	Targeted Therapy
TLA ATG	Chemotherapy

Trabectedin	Chemotherapy
Trametinib	Targeted Therapy
Trastuzumab	Targeted Therapy
Tretinoin Arsenic	Targeted Therapy
Trifluridine Tiparacil	Chemotherapy
Triptorelin Decapeptyl Sr	Hormone Therapy
Venetoclax	Targeted Therapy
Vinblastine	Chemotherapy
Vincristine	Chemotherapy
Vinorelbine	Chemotherapy
Zoledronic Acid	Supportive Treatment
Zometa	Supportive Treatment

© 2022 Várnai C et al. *JAMA Network Open.*

e**Table 2.** Basic Patient Information Within 4 Categories: All, Patients Receiving Chemotherapy, Patients Not Receiving Chemotherapy (Including Not Receiving Treatment), and Patients Not Receiving Treatment

	**All patients**			**Chemotherapy**			**Not on Chemotherapy**			**Not on Treatment**		
**Set**	**All**	**Died**	**Survived**	**All**	**Died**	**Survived**	**All**	**Died**	**Survived**	**All**	**Died**	**Survived**
	2515	966 (38%)	1549 (62%)	587 (23%)	169 (29%)	418 (71%)	1928 (77%)	797 (41%)	1131 (59%)	1135 (45%)	513 (45%)	622 (55%)
**Patient Characteristic**												
Female	1051 (42%)	343 (36%)	708 (46%)	302 (51%)	65 (38%)	237 (57%)	749 (39%)	278 (35%)	471 (42%)	431 (38%)	185 (36%)	246 (40%)
Male	1464 (58%)	623 (64%)	841 (54%)	285 (49%)	104 (62%)	181 (43%)	1179 (61%)	519 (65%)	660 (58%)	704 (62%)	328 (64%)	376 (60%)
Age	71.9 (62-80)	75 (66-82)	70 (59-77)	65 (56-73)	70 (62-76)	64 (54-72)	73 (64-81)	76 (67-83)	72 (62-80)	74 (65-82)	76.4 (67.6-84)	73 (62.2-80.9)
BMI	26.2 (22.6-30.5)	26.1 (22.9-31.4)	26.2 (22.4-30.1)	26.3 (22.7-30.4)	26.1 (23.6-30.1)	26.4 (22.5-30.5)	26.1 (22.5-30.5)	26.1 (22.8-31.4)	26.1 (22.4-29.7)	24.9 (21.8-29.4)	25.5 (22.2-30.9)	24.6 (21.5-29)
**Ethnicity**												
BAME	272 (11%)	106 (11%)	166 (11%)	61 (10%)	17 (10%)	44 (11%)	211 (11%)	89 (11%)	122 (11%)	124 (11%)	52 (10%)	72 (12%)
White	1487 (59%)	571 (59%)	916 (59%)	352 (60%)	113 (67%)	239 (57%)	1135 (59%)	458 (57%)	677 (60%)	658 (58%)	289 (56%)	369 (59%)
No information	756 (30%)	289 (30%)	467 (30%)	174 (30%)	39 (23%)	135 (32%)	582 (30%)	250 (31%)	332 (29%)	353 (31%)	172 (34%)	181 (29%)
**Comorbidities**												
Cardiovascular disease	489 (19%)	242 (25%)	247 (16%)	64 (11%)	23 (14%)	41 (10%)	425 (22%)	219 (27%)	206 (18%)	273 (24%)	144 (28%)	129 (21%)
COPD	263 (10%)	125 (13%)	138 (9%)	40 (7%)	16 (9%)	24 (6%)	223 (12%)	109 (14%)	114 (10%)	141 (12%)	75 (15%)	66 (11%)
Hypertension	928 (37%)	409 (42%)	519 (34%)	151 (26%)	49 (29%)	102 (24%)	777 (40%)	360 (45%)	417 (37%)	458 (40%)	223 (43%)	235 (38%)
Vascular	235 (9%)	110 (11%)	125 (8%)	24 (4%)	9 (5%)	15 (4%)	211 (11%)	101 (13%)	110 (10%)	123 (11%)	58 (11%)	65 (10%)
CKD	243 (10%)	133 (14%)	110 (7%)	19 (3%)	13 (8%)	6 (1%)	224 (12%)	120 (15%)	104 (9%)	157 (14%)	90 (18%)	67 (11%)
Diabetes	489 (19%)	216 (22%)	273 (18%)	70 (12%)	28 (17%)	42 (10%)	419 (22%)	188 (24%)	231 (20%)	256 (23%)	124 (24%)	132 (21%)
Other	1064 (42%)	437 (45%)	627 (40%)	195 (33%)	61 (36%)	134 (32%)	869 (45%)	376 (47%)	493 (44%)	550 (48%)	254 (50%)	296 (48%)
No key comorbidity	1052 (42%)	312 (32%)	740 (48%)	347 (59%)	80 (47%)	267 (64%)	705 (37%)	232 (29%)	473 (42%)	385 (34%)	147 (29%)	238 (38%)
**Solid Organ Cancer Types**												
Breast (C50)	291 (12%)	71 (7%)	220 (14%)	84 (14%)	8 (5%)	76 (18%)	207 (11%)	63 (8%)	144 (13%)	71 (6%)	31 (6%)	40 (6%)
Prostate (C61)	272 (11%)	117 (12%)	155 (10%)	13 (2%)	7 (4%)	6 (1%)	259 (13%)	110 (14%)	149 (13%)	117 (10%)	42 (8%)	75 (12%)
Lung (C34)	265 (11%)	131 (14%)	134 (9%)	52 (9%)	23 (14%)	29 (7%)	213 (11%)	108 (14%)	105 (9%)	130 (11%)	74 (14%)	56 (9%)
Colorectal (C18-C21)	253 (10%)	86 (9%)	167 (11%)	75 (13%)	17 (10%)	58 (14%)	178 (9%)	69 (9%)	109 (10%)	124 (11%)	58 (11%)	66 (11%)
Digestive organs (non-colorectal) (C15-C17, C22-C26)	200 (8%)	75 (8%)	125 (8%)	67 (11%)	13 (8%)	54 (13%)	133 (7%)	62 (8%)	71 (6%)	103 (9%)	56 (11%)	47 (8%)
Urinary tract (C64-C68)	172 (7%)	78 (8%)	94 (6%)	12 (2%)	5 (3%)	7 (2%)	160 (8%)	73 (9%)	87 (8%)	98 (9%)	49 (10%)	49 (8%)
Melanoma (C43-C44)	130 (5%)	46 (5%)	84 (5%)	0 (0%)	0 (0%)	0 (0%)	130 (7%)	46 (6%)	84 (7%)	79 (7%)	31 (6%)	48 (8%)
Female genital organs (C51-C58)	107 (4%)	32 (3%)	75 (5%)	41 (7%)	5 (3%)	36 (9%)	66 (3%)	27 (3%)	39 (3%)	45 (4%)	20 (4%)	25 (4%)
Lip, oral cavity and pharynx (C00-C14)	55 (2%)	15 (2%)	40 (3%)	17 (3%)	1 (1%)	16 (4%)	38 (2%)	14 (2%)	24 (2%)	22 (2%)	11 (2%)	11 (2%)
CNS (C69-C72)	52 (2%)	21 (2%)	31 (2%)	14 (2%)	4 (2%)	10 (2%)	38 (2%)	17 (2%)	21 (2%)	29 (3%)	15 (3%)	14 (2%)
Other solid organ cancers	114 (5%)	32 (3%)	82 (5%)	31 (5%)	6 (4%)	25 (6%)	83 (4%)	26 (3%)	57 (5%)	54 (5%)	19 (4%)	35 (6%)
**Haematological Cancer Types**												
Lymphoma (C81-C85 C88)	231 (9%)	89 (9%)	142 (9%)	92 (16%)	34 (20%)	58 (14%)	139 (7%)	55 (7%)	84 (7%)	102 (9%)	38 (7%)	64 (10%)
Myeloma and Plasmacytoma (C.90)	134 (5%)	66 (7%)	68 (4%)	22 (4%)	12 (7%)	10 (2%)	112 (6%)	54 (7%)	58 (5%)	33 (3%)	12 (2%)	21 (3%)
Acute leukemia and MDS (C91.0, C92.0, D46)	89 (4%)	45 (5%)	44 (3%)	46 (8%)	23 (14%)	23 (6%)	43 (2%)	22 (3%)	21 (2%)	38 (3%)	18 (4%)	20 (3%)
CLL (C91.1)	68 (3%)	27 (3%)	41 (3%)	5 (1%)	3 (2%)	2 (0%)	63 (3%)	24 (3%)	39 (3%)	43 (4%)	17 (3%)	26 (4%)
Other mature lymphoid leukemia (C91.3-C91.6)	42 (2%)	15 (2%)	27 (2%)	8 (1%)	3 (2%)	5 (1%)	34 (2%)	12 (2%)	22 (2%)	24 (2%)	10 (2%)	14 (2%)
Other haematological cancers	40 (2%)	20 (2%)	20 (1%)	8 (1%)	5 (3%)	3 (1%)	32 (2%)	15 (2%)	17 (2%)	23 (2%)	12 (2%)	11 (2%)
**Cancer Stage**												
Tumour with metastasis	959 (38%)	400 (41%)	559 (36%)	239 (41%)	73 (43%)	166 (40%)	720 (37%)	327 (41%)	393 (35%)	369 (33%)	199 (39%)	170 (27%)
Primary locally advanced tumour	296 (12%)	99 (10%)	197 (13%)	88 (15%)	15 (9%)	73 (17%)	208 (11%)	84 (11%)	124 (11%)	128 (11%)	60 (12%)	68 (11%)
Primary localized tumour	503 (20%)	150 (16%)	353 (23%)	83 (14%)	12 (7%)	71 (17%)	420 (22%)	138 (17%)	282 (25%)	260 (23%)	93 (18%)	167 (27%)
No information	757 (30%)	317 (33%)	440 (28%)	177 (30%)	69 (41%)	108 (26%)	580 (30%)	248 (31%)	332 (29%)	378 (33%)	161 (31%)	217 (35%)
**COVID-19 Severity**												
Asymptomatic	119 (5%)	15 (2%)	104 (7%)	35 (6%)	3 (2%)	32 (8%)	84 (4%)	12 (2%)	72 (6%)	48 (4%)	5 (1%)	43 (7%)
Mild	1108 (44%)	138 (14%)	970 (63%)	328 (56%)	31 (18%)	297 (71%)	780 (40%)	107 (13%)	673 (60%)	421 (37%)	76 (15%)	345 (55%)
Severe	701 (28%)	302 (31%)	399 (26%)	121 (21%)	45 (27%)	76 (18%)	580 (30%)	257 (32%)	323 (29%)	362 (32%)	170 (33%)	192 (31%)
Critical	539 (21%)	493 (51%)	46 (3%)	97 (17%)	87 (51%)	10 (2%)	442 (23%)	406 (51%)	36 (3%)	278 (24%)	252 (49%)	26 (4%)
No information	48 (2%)	18 (2%)	30 (2%)	6 (1%)	3 (2%)	3 (1%)	42 (2%)	15 (2%)	27 (2%)	26 (2%)	10 (2%)	16 (3%)

© 2022 Várnai C et al. *JAMA Network Open.*

Absolute numbers and percentages given, including sub-divisions by patient survival (died, survived). Percentages in a category refer to the absolute number of patients within a category, percentages in the subdivision 'died' and 'not died' refer to the number of patients in the respective sub-category, and percentages in the header to the total number of patients. Where appropriate, median, upper, and lower quartile are given for numeric values.

© 2022 Várnai C et al. *JAMA Network Open.*

eTable 3. Association of Comorbidities on Patient Outcome for All Patients, All-Cause Death to Survive (Including Remaining in Hospital), Using Patients With Valid Outcome, Age, Sex, and Comorbidities

		**Multivariate**	
**comorbidity**	**N**	**OR**	**P**
Cardiovascular	489	1.26 (1.03,1.55)	0.024
CKD	243	1.58 (1.19,2.09)	1.69E-03
COPD	263	1.28 (0.94,1.75)	0.117
Diabetes	489	1.18 (0.92,1.51)	0.197
Hypertension	928	1.15 (0.95,1.38)	0.143
Vascular	235	1.23 (0.97,1.55)	0.088
Other	920	1.08 (0.91,1.28)	0.379
None	737	0.78 (0.62,0.99)	0.044
At least 1 co-morbidity	1463	1.41 (1.13,1.75)	2.30E-03
At least 2 co-morbidities	764	1.39 (1.13,1.73)	2.30E-03

N: number of patients with the comorbidity. OR: odds ratio and 95% confidence interval in multivariate analysis adjusted for age and sex but not the other comorbidities. P: nominal P-value for this multivariate analysis. When all patients were included CVD (OR 1.26; 95% CI 1.03-1.55) and CKD (OR 1.58; 95% CI 1.19-2.09) were associated with a significantly increased risk of death. In solid cancer patients, CVD, CKD, COPD and DM were associated with a significantly increased risk of death whilst in haematological cancer patients, only CKD was significantly associated with an increased risk of death in multivariate analysis adjusting for age and sex only (OR 1.81, 1.10-2.97).

© 2022 Várnai C et al. *JAMA Network Open.*

eTable 4. Association of Anticancer Treatment in the 4 Weeks Prior to COVID-19 Diagnosis With All-Cause Mortality

Comparison	N	OR	P	OR_mv	P_mv
**Anticancer treatment within 4 weeks of COVID-19 diagnosis**					
Chemotherapy vs no chemotherapy	587	0.57 (0.47-0.70)	3.11E-08	0.82 (0.62-1.07)	0.146
Radiotherapy vs no radiotherapy	175	0.85 (0.60-1.18)	0.334	0.90 (0.64-1.28)	0.560
Hormone therapy vs no hormone therapy	250	0.91 (0.69-1.20)	0.538	0.74 (0.54-1.01)	0.058
targeted therapy vs no targeted therapy	255	0.77 (0.58-1.02)	0.067	0.97 (0.72-1.31)	0.859
surgery vs no surgery	93	0.58 (0.35-0.94)	0.022	0.55 (0.32-0.93)	0.027
Immunotherapy (CPI) vs no immunotherapy (CPI)	102	0.57 (0.35-0.90)	0.012	0.62 (0.37-1.02)	0.061
immunomodulatory vs no immunomodulatory	58	2.16 (1.23-3.81)	4.04E-03	2.22 (1.25-3.93)	6.46E-03
**Anticancer treatment within 4 weeks of COVID-19 diagnosis, compared to no treatment**					
Chemotherapy vs no treatment	587	0.49 (0.39-0.61)	3.51E-11	0.70 (0.52-0.94)	0.018
Radiotherapy vs no treatment	175	0.65 (0.46-0.91)	0.011	0.74 (0.53-1.03)	0.072
Hormone therapy vs no treatment	250	0.69 (0.52-0.93)	0.011	0.66 (0.48-0.91)	0.012
targeted therapy vs no treatment	255	0.60 (0.44-0.80)	3.49E-04	0.80 (0.57-1.11)	0.175
surgery vs no treatment	93	0.45 (0.27-0.73)	6.81E-04	0.47 (0.28-0.78)	3.38E-03
Immunotherapy (CPI) vs no treatment	102	0.44 (0.27-0.70)	2.39E-04	0.52 (0.31-0.86)	0.011
Immunomodulatory vs no treatment	58	1.60 (0.91-2.85)	0.104	1.75 (1.00-3.07)	0.049
**Cytotoxic chemotherapy**					
non-palliative chemotherapy vs palliative chemotherapy	193	0.36 (0.22-0.57)	3.80E-06	0.44 (0.26-0.76)	3.04E-04
palliative first-line chemotherapy vs other lines	343	1.06 (0.67-1.71)	0.822	1.05 (0.59-1.86)	0.871
palliative chemotherapy vs no chemotherapy	268	0.85 (0.64-1.12)	0.245	1.07 (0.78-1.47)	0.690
palliative chemotherapy vs no treatment	268	0.72 (0.53-0.96)	0.025	0.89 (0.63-1.25)	0.491
**Radio therapy**					
non-palliative radio therapy vs palliative radio therapy	83	0.21 (0.09-0.46)	1.52E-05	0.22 (0.10-0.50)	3.25E-04
palliative first-line radio therapy vs other lines	113	0.76 (0.23-2.71)	0.584	0.85 (0.30-2.40)	0.758
palliative radio therapy vs no radio therapy	70	1.75 (1.05-2.91)	0.024	1.70 (1.06-2.74)	0.029
palliative radio therapy vs no treatment	70	1.31 (0.78-2.21)	0.315	1.36 (0.90-2.05)	0.139
**Hormone therapy**					
non-palliative hormone therapy vs palliative hormone therapy	53	0.66 (0.31-1.35)	0.245	0.75 (0.34-1.64)	0.466
palliative first-line hormone therapy vs other lines	115	0.87 (0.44-1.75)	0.742	0.80 (0.40-1.62)	0.542
palliative hormone therapy vs no hormone therapy	136	1.10 (0.76-1.60)	0.581	0.92 (0.61-1.39)	0.692

© 2022 Várnai C et al. *JAMA Network Open.*

palliative hormone therapy vs no treatment	136	0.82 (0.55-1.20)	0.302	0.81 (0.54-1.22)	0.309
**Targeted therapy**					
non-palliative targeted therapy vs palliative targeted therapy	31	0.47 (0.15-1.29)	0.137	0.63 (0.23-1.70)	0.360
palliative first-line targeted therapy vs other lines	101	0.56 (0.28-1.11)	0.077	0.58 (0.34-0.99)	0.045
palliative targeted therapy vs no targeted therapy	139	0.83 (0.57-1.22)	0.362	1.03 (0.71-1.50)	0.866
palliative targeted therapy vs no treatment	139	0.63 (0.42-0.94)	0.020	0.81 (0.52-1.26)	0.340
**Surgery**					
non-palliative surgery vs palliative surgery	61	0.88 (0.08-47.23)	1.000	0.46 (0.05-4.10)	0.485
palliative first-line surgery vs other lines	45	0.56 (0.04-33.11)	0.522	0.32 (0.02-5.49)	0.432
**Immunotherapy(CPI)**					
palliative first-line immunotherapy (CPI) vs other lines	50	1.56 (0.48-5.68)	0.595	2.33 (0.60-9.08)	0.224
palliative immunotherapy (CPI) vs no immunotherapy (CPI)	68	0.74 (0.41-1.27)	0.307	0.73 (0.42-1.29)	0.279
palliative immunotherapy (CPI) vs no treatment	68	0.55 (0.31-0.97)	0.030	0.60 (0.34-1.04)	0.066
**Immunomodulatory**					
palliative first-line immunomodulatory therapy vs other lines	16	0.79 (0.19-3.31)	0.757	0.74 (0.19-2.89)	0.670
palliative immunomodulatory therapy vs no immunomodulatory therapy	38	2.11 (1.05-4.30)	0.027	1.99 (1.05-3.79)	0.036
palliative immunomodulatory therapy vs no treatment	38	1.53 (0.76-3.15)	0.242	1.57 (0.83-2.99)	0.168

N: number of patients; OR: odds ratio and 95% confidence interval of a univariate analysis. P: univariate nominal P-value. OR_mv: odds ratio and 95% confidence interval in a multivariate analysis adjusted for age, sex, and comorbidities; P_mv: nominal P-value for this multivariate analysis.

eTable 5. Association of Multiple Treatment Types on All-Cause Mortality

Comparison	Reference	Cancer types	N_all	D_all	N	D	OR	P	OR_mv	P_mv
palliative chemotherapy + SACT	palliative chemotherapy only	all	216	77	48	18	1.11 (0.53-0.53)	0.864	1.01 (0.57-1.78)	0.970
palliative chemotherapy + SACT	palliative chemotherapy only	solid organ	184	56	37	11	0.96 (0.39-0.39)	1.000	0.94 (0.48-1.83)	0.853
palliative chemotherapy + SACT	palliative chemotherapy only	haemato-logical	32	21	11	7	0.88 (0.15-0.15)	1.000	1.05 (0.13-8.59)	0.963
chemotherapy multiple-agent	chemotherapy single-agent	solid organ	265	56	132	33	1.59 (0.84-0.84)	0.135	1.55 (0.75-3.19)	0.234

N_all: number of patients with a COVID diagnosis. D_all: number of patients with a COVID diagnosis who died. N: number of patients in the comparison group with a COVID diagnosis. D: number of patients in the comparison group with a COVID diagnosis who died. OR: odds ratio and 95% confidence interval of a univariate analysis comparing the comparison group to those in the reference group. P: univariate nominal P-value. OR_mv: odds ratio and 95% confidence interval in a multivariate analysis adjusted for age, sex and comorbidities. P_mv: nominal P-value for this multivariate analysis.

© 2022 Várnai C et al. *JAMA Network Open.*

eTable 6. Association of Recent Immunotherapy (in the 4 Weeks Before a COVID-19 Diagnosis) With All-Cause Mortality for Individual Cancer Types

**Immunotherapy vs no immunotherapy**									
**Cancer subtype**	**N**	**N_d**	**N_i**	**N_di**	**OR**	**P**	**OR_mv**	**P_mv**	**Fatality**
Melanoma (C43-C44)	130	46	31	6	0.36 (0.11-1.00)	0.034	0.87 (0.22-3.35)	0.834	0.194
Urinary tract (C64-C68)	172	78	18	6	0.57 (0.17-1.75)	0.325	0.68 (0.21-2.22)	0.523	0.333
Lung (C34)	265	131	43	15	0.49 (0.23-1.01)	0.045	0.55 (0.26-1.16)	0.117	0.349
**Immunotherapy vs no treatment**									
**Cancer subtype**	**N**	**N_d**	**N_i**	**N_di**	**OR**	**P**	**OR_mv**	**P_mv**	**Fatality**
Melanoma (C43-C44)	110	37	31	6	0.37 (0.11-1.08)	0.072	0.87 (0.20-3.76)	0.857	0.194
Urinary tract (C64-C68)	116	55	18	6	0.50 (0.14-1.59)	0.212	0.58 (0.15-2.17)	0.415	0.333
Lung (C34)	173	89	43	15	0.41 (0.18-0.88)	0.014	0.46 (0.20-1.06)	0.069	0.349

All patients with a valid all-cause-death outcome, cancer type, cancer treatment information, age, sex and comorbidities were included. Cancer subtypes are given with ICD-10 codes (International Classification of Diseases, 10th Revision); N: number of patients with the cancer type. N_d: number of patients with the cancer type who died. N_i: number of patients with the cancer type who received immunotherapy in the past 4 weeks. N_di: number of patients with the cancer type who received immunotherapy in the past 4 weeks and died. OR: odds ratio and 95% confidence interval of a univariate analysis comparing patients receiving immunotherapy and those who did not. P: univariate nominal P-value. OR_mv: odds ratio and 95% confidence interval in a multivariate analysis adjusted for age, sex and comorbidities. P_mv: nominal P-value for this multivariate analysis; Fatality: Case fatality rate.

eTable 7. Association of Cancer Treatments With All-Cause Mortality for Patients With Lung Cancer

**Comparing treatment to other treatment / no treatment**						
**treatment**	**N**	**D**	**OR**	**P**	**OR_mv**	**P_mv**
chemotherapy vs no chemotherpy	52	23	0.77 (0.40-1.48)	0.441	0.82 (0.43-1.54)	0.531
Immunotherapy (CPI) vs no immunotherapy (CPI)	43	15	0.49 (0.23-1.01)	0.045	0.55 (0.26-1.16)	0.117
targeted therapy vs no targeted therapy	18	8	0.81 (0.27-2.36)	0.808	0.97 (0.36-2.63)	0.955
radiotherapy vs no radiotherapy	37	17	0.85 (0.40-1.81)	0.724	0.90 (0.43-1.88)	0.779
Chemotherapy + immunotherapy (CPI) vs no chemotherapy + immunotherapy (CPI)	16	6	0.60 (0.17-1.88)	0.441	0.68 (0.21-2.20)	0.522
**Comparing treatment to no cancer treatment**						
**treatment**	**N**	**D**	**OR**	**P**	**OR_mv**	**P_mv**
chemotherapy vs no treatment	52	23	0.60 (0.30-1.21)	0.140	0.62 (0.30-1.30)	0.208
Immunotherapy (CPI) vs no treatment	43	15	0.41 (0.18-0.88)	0.014	0.46 (0.20-1.06)	0.069
targeted therapy vs no treatment	18	8	0.61 (0.19-1.84)	0.327	0.76 (0.25-2.32)	0.632
radiotherapy vs no treatment	37	17	0.64 (0.29-1.43)	0.265	0.66 (0.30-1.44)	0.297
Chemotherapy + immunotherapy (CPI) vs no treatment	16	6	0.46 (0.13-1.48)	0.185	0.52 (0.14-1.91)	0.326

Treatments or treatment combinations considered were administered to at least 10 lung cancer patients. *N: number of patients who received the cancer treatment in the past 4 weeks. D: number of patients who received the cancer treatment in the past 4 weeks and died. OR: odds ratio and 95% confidence interval of a univariate analysis comparing patients receiving the treatment and those who did not. P: univariate nominal P-value. OR_mv: odds ratio and 95% confidence interval in a multivariate analysis adjusted for age, sex and comorbidities. P_mv: nominal P-value for this multivariate analysis.*

© 2022 Várnai C et al. *JAMA Network Open.*

eTable 8. Association of the Time Difference Between the Last Cancer Treatment and COVID-19 Diagnosis With All-Cause Mortality

**comparison**	**N_all**	**N_all_died**	**N**	**N_died**	**OR**	**P**	**OR_mv**	**P_mv**
0-2wk vs 2-12wk	531	213	256	105	1.08 (0.75-1.54)	0.723	1.11 (0.80-1.55)	0.518
0-2wk vs 2-4wk	380	146	256	105	1.41 (0.88-2.27)	0.145	1.49 (0.91-2.44)	0.112
0-2wk vs 2-4wk, chemo only	183	60	114	40	1.32 (0.66-2.69)	0.421	1.71 (0.79-3.72)	0.176
0-4wk vs 4-12wk	531	213	380	146	0.78 (0.52-1.17)	0.239	0.81 (0.56-1.18)	0.270
**comparison**	**N_all**	**N_all_died**	**N**	**N_died**	**OR**	**P**	**OR_mv**	**P_mv**
0-3wk vs 3-12wk	531	213	329	126	0.82 (0.57-1.19)	0.316	0.85 (0.58-1.23)	0.383
0-3wk vs 3-6wk	445	170	329	126	1.02 (0.64-1.61)	1.000	1.03 (0.66-1.63)	0.886
0-6wk vs 6-12wk	531	213	445	170	0.62 (0.38-1.01)	0.054	0.65 (0.42-1.00)	0.053

N_all: number of patients with either time difference compared. N_all_died: number of patients with either time difference compared who died. N: number of patients with the time difference listed first. N_died: number of patients with the time difference listed first who died. OR: odds ratio and 95% confidence interval of a univariate analysis comparing patients treated in the time difference listed first compared to those treated in the time difference listed second. P: univariate nominal P-value. OR_mv: odds ratio and 95% confidence interval in a multivariate analysis adjusted for age, sex and comorbidities. P_mv: nominal P-value for this multivariate analysis.

© 2022 Várnai C et al. *JAMA Network Open.*

eTable 9. Association of Blood Count Levels of CRP, Neutrophils, and Lymphocytes With All-Cause Mortality

**CRP - too high**								
**Patients**	**Blood count**	**Reference**	**N**	**D**	**OR**	**P**	**OR_mv**	**P_mv**
All	>=82 (median)	<82	1113	445	3.00 (2.32-3.88)	2.23E-18	2.76 (2.18-3.48)	1.26E-17
Solid organ	>=81 (median)	<81	782	304	3.37 (2.47-4.63)	8.22E-16	3.14 (2.40-4.10)	8.85E-17
hematological	>=84 (median)	<84	331	141	2.61 (1.63-4.21)	2.44E-05	2.31 (1.46-3.64)	3.29E-04
**Neutrophils - too high**								
**Patients**	**Blood count**	**Reference**	**N**	**D**	**OR**	**P**	**OR_mv**	**P_mv**
Solid organ	>8	>=2 and <=8	899	351	2.96 (2.19-4.00)	2.12E-13	3.15 (2.08-4.76)	5.80E-08
hematological	>8	>=2 and <=8	252	106	2.80 (1.45-5.49)	1.09E-03	2.66 (1.41-5.04)	2.66E-03
**Neutrophils - too low**								
**Patients**	**Blood count**	**Reference**	**N**	**D**	**OR**	**P**	**OR_mv**	**P_mv**
Solid organ	<2	>=2 and <=8	693	208	0.71 (0.39-1.25)	0.242	1.03 (0.54-1.95)	0.927
hematological	<2	>=2 and <=8	321	125	1.29 (0.80-2.10)	0.291	1.54 (0.96-2.46)	0.072
**Lymphocytes - too low compared to normal range (1-4)**								
**Patients**	**Blood count**	**Reference**	**N**	**D**	**OR**	**P**	**OR_mv**	**P_mv**
Solid organ	<1	>=1 and <=4	536	188	1.95 (1.34-2.85)	2.98E-04	1.79 (1.26-2.55)	1.19E-03
hematological	<1	>=1 and <=4	234	97	1.93 (1.06-3.57)	0.025	1.96 (1.25-3.07)	3.32E-03
**N/L ratio**								
**Patients**	**Blood count**	**Reference**	**N**	**D**	**OR**	**P**	**OR_mv**	**P_mv**
Solid organ	>=7.02 (median)	<7.02	978	370	2.61 (1.98-3.45)	1.31E-12	2.38 (1.81-3.12)	4.31E-10
hematological	>=4.5 (median)	<4.5	373	159	1.80 (1.16-2.78)	6.45E-03	1.58 (1.06-2.35)	0.023

Blood count: patients with these blood count values are compared. Reference: blood count values defining the reference group of patients. N: number of patients with the blood count level compared. D: number of patients with the blood count level compared who died. OR: odds ratio and 95% confidence interval of a univariate analysis comparing patients with the blood count compared to the reference. P: univariate nominal P-value. OR_mv: odds ratio and 95% confidence interval in a multivariate analysis adjusted for age, sex and comorbidities. P_mv: nominal P-value for this multivariate analysis.

© 2022 Várnai C et al. *JAMA Network Open.*

eTable 10. Patient Information on Admission to Intensive Care Unit

**ITU admission and ventilation**									
**cancer types**	**all**			**ITU admission (of all)**		**non-invasive ventilation (of ITU admitted)**		**invasive ventilation (of not ITU admitted)**	
all	2515	-	-	131	5.2%	94	71.8%	72	3.0%
**ITU admission of patients with curative treatment intent**									
**cancer types**	**all (with treatment intent)**	**curative (of all)**		**ITU admission (of curative)**		**died (of curative)**		**ITU admission (of died, curative)**	
all	1463	541	37.0%	37	6.8%	126	23.3%	22	17.5%
solid organ	1145	403	35.2%	19	4.7%	78	19.4%	9	11.5%
haematological	318	138	43.4%	18	13.0%	48	34.8%	13	27.1%

Absolute numbers and percentages given. Percentages are of the total number indicated in the header, either percentage of all, percentage admitted to ITU, percentage not admitted to ITU, percentage of curative or percentage of those that died.

© 2022 Várnai C et al. *JAMA Network Open.*

eTable 11. Comparison of the Patients Included in the CCC-19 Cohort [3] and This Study (UKCCMP)

Patients	CCC-19[a]	UKCCMP[b]
All	4966	-
Died	695 (14.0%)	-
With active cancer, all	2420	-
With active cancer, died	426 (17.6%)	-
Hospitalized, all	2872	2515
Hospitalized, died	673 (23.4%)	966 (38.4%)
Hospitalized, with active cancer, all	1506	2445
Hospitalized, with active cancer, died	411 (27.3%)	950 (38.9%)

Number of patients and percentages of all-cause deaths are given compared to all patients in the same category.
[a]: including patients for whom data were imputed; [b]: number of patients for whom age, sex, comorbidity and cancer type data were complete. No data imputation was applied.

© 2022 Várnai C et al. *JAMA Network Open.*

eReferences

1. WHO. International Statistical Classification of Diseases and Related Health Problems 10th Revision.
2. Clinical management of severe acute respiratory infection when novel coronavirus (2019-nCoV) infection is suspected: interim guidance, 28 January 2020. . 2020. at https://apps.who.int/iris/handle/10665/330893.)
3. Grivas P, Khaki AR, Wise-Draper TM, et al. Association of clinical factors and recent anticancer therapy with COVID-19 severity among patients with cancer: a report from the COVID-19 and Cancer Consortium. Ann Oncol 2021;32:787-800.

© 2022 Várnai C et al. *JAMA Network Open.*
