# Supplementary material for: Mortality Among Adults With Cancer Undergoing Chemotherapy or Immunotherapy and Infected With COVID-19
Source: JAMA Netw Open. 2022 Feb 21;5(2):e220130. doi: 10.1001/jamanetworkopen.2022.0130 (PMC8861846; doi:10.1001/jamanetworkopen.2022.0130)
Supplement: Supplement 2. — UKCCMP Team Members [file jamanetwopen-e220130-s002.pdf]

Supplemental Online Content: Nonauthor Collaborators

*Indicates required information. Only first name, last name, and suffix will appear in PubMed.

*Group Name(s): UKCCMP Team							
***First Name and Middle Initial(s)**	***Last Name**	***Suffix (eg, Jr, III)**	Academic Degrees	Institution	Location (city, state/province, country)	Role or Contribution, eg, chair, principal investigator	Group (if more than 1 Group listed in the byline) and/or Subgroup (eg, Steering Committee)
Sian	Pugh			Addenbrooke's Hospital	Cambridge, UK		
Pippa	Corrie			Addenbrooke's Hospital	Cambridge, UK		
Ahmed	Bedair			Altnagelvin Hospital - North West Cancer Centre	Londonderry, UK		
Madeleine	Hewish			Ashford and St Peters Hospital	Ashford, UK		
Pauline	Leonard			Barking, Havering And Redbridge University Hospitals NHS Trust	UK		
Jack	Illingworth			Barking, Havering And Redbridge University Hospitals NHS Trust	UK		
Stephen	Hibbs			Barking, Havering And Redbridge University Hospitals NHS Trust	UK		
Nikolaos	Diamantis			Barts Health NHS Trust	UK		
Samah	Massalha			Betsi Cadwaladr University Health Board	UK		
Claire	Fuller			Betsi Cadwaladr University Health Board	UK		
Caroline	Usborne			Betsi Cadwaladr University Health Board	UK		
Duncan	Gilbert			Brighton And Sussex Medical School, University of Sussex	UK		
Jennifer	Davies			Buckinghamshire Healthcare NHS Trust	UK		
Tom	Newsom-Davis			Buckinghamshire Healthcare NHS Trust	UK		
Rachel	Sharkey			Chelsea And Westminster Hospital NHS Foundation Trust	UK		
Rebecca	Lee			The Christie NHS Foundation Trust	UK		
Ann	Tivey			The Christie NHS Foundation Trust	UK		
Rohan	Shotton			The Christie NHS Foundation Trust	UK		

*Indicates required information. Only first name, last name, and suffix will appear in PubMed.

*First Name and Middle Initial(s)	*Last Name	*Suffix (eg, Jr, III)	Academic Degrees	Institution	Location (city, state/province, country)	Role or Contribution, eg, chair, principal investigator	Group (if more than 1 Group listed in the byline) and/or Subgroup (eg, Steering Committee)
Clare	Griffin			The Christie NHS Foundation Trust	UK		
Laura	Horsley			The Christie NHS Foundation Trust	UK		
Simon	Shamas			The Clatterbridge Cancer Centre	Wirral, UK		
Joseph J	Sacco			The Clatterbridge Cancer Centre	Wirral, UK		
Madbuba	Choudhury			Colchester University Hospital	UK		
Jillian	Noble			Croydon University Hospital	UK		
Heather	Shaw			East And North Hertfordshire NHS Trust	UK		
Rachel	Bolton			East Lancashire Hospitals NHS Trust	UK		
Ana	Ferreira			East Lancashire Hospitals NHS Trust	UK		
Peter	Hall			Edinburgh Cancer Centre, University of Edinburgh	UK		
Paul	Ramage			Edinburgh Cancer Centre, University of Edinburgh	UK		
Jaishree	Bhosle			Epsom And St Helier University Hospitals	UK		
Alison	Massey			Epsom And St Helier University Hospitals	UK		
Michaela	Hill			George Eliot Hospital NHS Trust	UK		
Leena	Mukherjee			Beatson West Of Scotland Cancer Centre	Glasgow, UK		
Aisha	Ghaus			Beatson West Of Scotland Cancer Centre	Glasgow, UK		
Sarah	Derby			Beatson West Of Scotland Cancer Centre	Glasgow, UK		
Sean	Brown			Gloucestershire Hospitals NHS Foundation Trust	UK		
Sarah	Lowndes			Great Western Hospitals NHS Foundation Trust	UK		

*Indicates required information. Only first name, last name, and suffix will appear in PubMed.

*First Name and Middle Initial(s)	*Last Name	*Suffix (eg, Jr, III)	Academic Degrees	Institution	Location (city, state/province, country)	Role or Contribution, eg, chair, principal investigator	Group (if more than 1 Group listed in the byline) and/or Subgroup (eg, Steering Committee)
Saoirse	Dolly			Guy's and St Thomas's NHS Foundation Trust	UK		
Beth	Russell			Guy's and St Thomas's NHS Foundation Trust	UK		
Charlotte	Moss			Guy's and St Thomas's NHS Foundation Trust	UK		
Daniel	Muller			Hampshire Hospitals NHS Foundation Trust	UK		
Annet	Pillai			Hull And East Yorkshire NHS Trust	UK		
Shakeel	Lowe			Hull And East Yorkshire NHS Trust	UK		
Lucy	Cook			Imperial College Healthcare NHS Trust	UK		
Christopher	Scrase			Ipswich Hospital	Ipswich, UK		
Rema	Jyothirmayi			Kent Oncology Centre	Maidstone, UK		
Ruth	Board			Lancashire Teaching Hospitals NHS Foundation Trust	UK		
Stephanie	Cornthwaite			Lancashire Teaching Hospitals NHS Foundation Trust	UK		
Shefali	Parikh			Leighton Hospital	Crewe, UK		
Emma	Cattell			Leeds Institute of Medical Research, University of Leeds	Leeds, UK		
Nicola	Cox			Leeds Institute of Medical Research, University of Leeds	Leeds, UK		
Abigail	Gault			Musgrove Park Hospital	Taunton, UK		
Sam	Moody			Musgrove Park Hospital	Taunton, UK		
Caroline	Dobeson			Musgrove Park Hospital	Taunton, UK		
Mark	Baxter			Northern Centre For Cancer Care	Newcastle, UK		
Tom	Roques			NHS Tayside	UK		
Alexander	Pawsey			NHS Tayside	UK		
Roderick	Oakes			Norfolk And Norwich University Hospital	Norwich, UK		

Supplemental Online Content: Nonauthor Collaborators

*Indicates required information. Only first name, last name, and suffix will appear in PubMed.

***First Name and Middle Initial(s)**	***Last Name**	***Suffix (eg, Jr, III)**	Academic Degrees	Institution	Location (city, state/province, country)	Role or Contribution, eg, chair, principal investigator	Group (if more than 1 Group listed in the byline) and/or Subgroup (eg, Steering Committee)
Lucinda	Melcher			North Cumbria Integrated Care NHS Foundation Trust			
Olivia	Chan			North Cumbria Integrated Care NHS Foundation Trust			
Sarah	Ayers			North Middlesex University Hospital,	London, UK		
Helen	Bowyer			North Middlesex University Hospital	London, UK		
Mohammed	Althohami			North West Anglia NHS Foundation Trust	UK		
Sajjan	Mittal			North West Anglia NHS Foundation Trust	UK		
Laura	Feeney			Northampton General Hospital NHS Trust	UK		
Avinash	Aujayeb			Northern Ireland Cancer Centre	Belfast, UK		
Omar	Sheikh			Northumbria Healthcare NHS Foundation Trust	UK		
Sangary	Kathirgamakarthigeyan			Northumbria Healthcare NHS Foundation Trust	UK		
Victoria	Woodcock			Nottingham University Hospitals NHS Trust	UK		
Francesca	Holt			Nottingham University Hospitals NHS Trust	UK		
Simon	Wyatt			Nottingham University Hospitals NHS Trust	UK		
Oliver	Topping			Oxford University Hospitals NHS Foundation Trust	UK		
Michael	Tilby			Oxford University Hospitals NHS Foundation Trust	UK		
Madhumita	Bhattacharyya			Queen Elizabeth Hospital Birmingham	UK		

Supplemental Online Content: Nonauthor Collaborators

*Indicates required information. Only first name, last name, and suffix will appear in PubMed.

*First Name and Middle Initial(s)	*Last Name	*Suffix (eg, Jr, III)	Academic Degrees	Institution	Location (city, state/province, country)	Role or Contribution, eg, chair, principal investigator	Group (if more than 1 Group listed in the byline) and/or Subgroup (eg, Steering Committee)
Emma	Burke			Queen Elizabeth Hospital Birmingham	UK		
Shawn	Ellis			Queen Elizabeth Hospital Birmingham	UK		
Joseph	Chacko			Royal Berkshire Hospital	Reading, UK		
Taslima	Rabbi			Royal Berkshire Hospital	Reading, UK		
Michael	Rowe			Royal Bournemouth Hospital	UK		
Rebecca	Sargent			Royal Bournemouth Hospital	UK		
Christina	Thirlwell			Royal Cornwall Hospital	UK		
Jack	Gibson			Royal Cornwall Hospital	UK		
Robert	Goldstein			Royal Devon And Exeter NHS Foundation Trust	UK		
Matthew	Fittall			Royal Free Hospital	UK		
Spyridon	Gennatas			Royal Free Hospital	UK		
Alicia	Okines			Royal Free Hospital	UK		
James	Best			Royal Marsden NHS Foundation Trust	UK		
Tania	Tillett			Royal Shrewsbury Hospital	UK		
Emily	Renninson			Royal Shrewsbury Hospital	UK		
Simon	Grumett			Royal United Hospitals Bath	UK		
Craig	Barrington			Royal Wolverhampton Hospital NHS Trust	UK		
Ruth	Pettengell			Singleton Hospital	Swansea, UK		
YingYing	Peng			Singleton Hospital	Swansea, UK		
Julia	Chackathayil			Singleton Hospital	Swansea, UK		
Akinfemi	Akingboye			St Georges University Hospital	London, UK		
Helen	Hollis			St Georges University Hospital	London, UK		
Ik Shin	Chin			The Dudley Group NHS Foundation	London, UK		
Vartika	Bisht			Institute Of Cancer And Genomic Sciences, University Of Birmingham	Birmingham, UK		

Supplemental Online Content: Nonauthor Collaborators

*Indicates required information. Only first name, last name, and suffix will appear in PubMed.

***First Name and Middle Initial(s)**	***Last Name**	***Suffix (eg, Jr, III)**	Academic Degrees	Institution	Location (city, state/province, country)	Role or Contribution, eg, chair, principal investigator	Group (if more than 1 Group listed in the byline) and/or Subgroup (eg, Steering Committee)
Simon	Hartley			Institute Of Cancer And Genomic Sciences, University Of Birmingham	Birmingham, UK		
Christopher P	Middleton			Institute Of Cancer And Genomic Sciences, University Of Birmingham	Birmingham, UK		
Anshita	Goel			The Dudley Group NHS Foundation Trust	UK		
Emily	Protheroe			Centre For Computational Biology, University Of Birmingham	Birmingham, UK		
Piangfan	Naksukpaiboon			University Of Birmingham Medical School, University Of Birmingham	Birmingham, UK		
Iris	Anil			The Dudley Group NHS Foundation Trust	Birmingham, UK		
Joel	Mitchell			Institute Of Cancer And Genomic Sciences, University Of Birmingham	Birmingham, UK		
Jamie	D'Costa			Centre For Computational Biology, University Of Birmingham	Birmingham, UK		
Anjui	Wu			UCL Cancer Institute, University College London	London, UK		
Diego	Ottaviani			UCL Cancer Institute, University College London	London, UK		
Gehan	Soosaipillai			UCL Cancer Institute, University College London	London, UK		
Myria	Galazi			UCL Cancer Institute, University College London	London, UK		
Neha	Chopra			UCL Cancer Institute, University College London	London, UK		
Sarah	Benafif			UCL Cancer Institute, University College London	London, UK		

*Indicates required information. Only first name, last name, and suffix will appear in PubMed.

*First Name and Middle Initial(s)	*Last Name	*Suffix (eg, Jr, III)	Academic Degrees	Institution	Location (city, state/province, country)	Role or Contribution, eg, chair, principal investigator	Group (if more than 1 Group listed in the byline) and/or Subgroup (eg, Steering Committee)
Heather	Shaw			UCL Cancer Institute, University College London	London, UK		
Christopher CT	Sng			UCL Cancer Institute, University College London	London, UK		
Sophia YN	Wong			UCL Cancer Institute, University College London	London, UK		
Martin	Scott-Brown			University Hospital Coventry And Warwickshire	UK		
Ellen	Copson			University Hospital Southampton NHS Foundation Trust	UK		
Timothy	Robinson			University Hospitals Bristol And Weston	UK		
Zoe	Hudson			University Hospitals Bristol And Weston	UK		
Fiona	Smith			University Hospitals Of Derby And Burton	UK		
Ali-Abdulnabi	Mohamed			University Hospitals of Leicester NHS Trust	UK		
Angelos	Angelakas			University Hospitals of Morecambe Bay NHS Foundation Trust	UK		
Leonie	Eastlake			University Hospitals Plymouth NHS Trust	UK		
Ashley	Poon-King			Velindre Cancer Centre	UK		
Clair	Brunner			Velindre Cancer Centre	UK		
Amy	Kwan			Weston Park Hospital	Sheffield, UK		
Alec	Maynard			Weston Park Hospital	Sheffield, UK		
Hayley	Boyce			Weston Park Hospital	Sheffield, UK		
Emma	Spurrell			Whittington Health NHS Trust	London, UK		
Rahul	Peck			Wigan, Wrightington And Leigh NHS Foundation Trust	UK		

*Indicates required information. Only first name, last name, and suffix will appear in PubMed.

***First Name and Middle Initial(s)**	***Last Name**	***Suffix (eg, Jr, III)**	Academic Degrees	Institution	Location (city, state/province, country)	Role or Contribution, eg, chair, principal investigator	Group (if more than 1 Group listed in the byline) and/or Subgroup (eg, Steering Committee)
Bartlomiej	Kurec			Worcester Royal Hospital	Worcester, UK		
